# Maturation Selection Biases and Relative Age Effect in Italian Soccer Players of Different Levels

**DOI:** 10.3390/biology11111559

**Published:** 2022-10-24

**Authors:** Stefania Toselli, Mario Mauro, Alessia Grigoletto, Stefania Cataldi, Luca Benedetti, Gianni Nanni, Riccardo Di Miceli, Paolo Aiello, Davide Gallamini, Francesco Fischetti, Gianpiero Greco

**Affiliations:** 1 Department for Life Quality Studies, University of Bologna, 47921 Rimini, Italy; 2 Department of Biomedical and Neuromotor Sciences, University of Bologna, 40126 Bologna, Italy; 3 Department of Translational Biomedicine and Neuroscience (DiBraiN), University of Study of Bari, 70124 Bari, Italy; 4 Isokinetic Research Center, 40132 Bologna, Italy; 5 Bologna F.C. 1909 Technical Center, 40128 Bologna, Italy; 6 Russi S. U., 48026 Ravenna, Italy

**Keywords:** maturation, soccer young players, physical skills, body composition

## Abstract

**Simple Summary:**

Soccer academies and societies research young players who are supposed to possess great motor skills. In association with these, adolescents who appear to be talented exhibit more developed anthropometric and body composition features than untalented players. Although many selected soccer players appear to present an earlier maturation than their contemporaries, it is not clear whether soccer teams of different competition levels are aware of this aspect and considered these discrepancies in the scouting selection process. In addition, it remains unclear if the biological maturity and relative effect are two interchangeable methods of evaluation and if one of them deserves to be preferred by the soccer scouters. This research aims to investigate the effect of the team level, maturity status and relative-age effect, and their interactions, on body characteristics, cellularity, and physical performance in adolescent soccer players. Despite the relative age effect remaining the easier analysis to consider, the evaluation of maturity status seems to be the most reliable method to detect better capabilities due to early growth.

**Abstract:**

Soccer is a sport practiced all over the world, in which players are expected to show specific physical and technical skills. Soccer academies look for young talented individuals to develop promising players. Although several parameters could affect the players’ performance, the relative age effect (RAE) and the maturity status appeared debated. Therefore, this study compared the differences in RAE and biological maturity among the players of two Italian soccer teams of different levels and to understand their interaction effects with the competition level on youth players’ physical characteristics and abilities. One hundred and sixty-two young soccer players from the U12 to U15 age categories of the elite (*n* = 98) and non-elite (*n* = 64) teams were recruited. The prevalence of maturity status and RAE was observed. Many anthropometric parameters, BIA vectors, and motor tests (CMJ, Sprint, RSA) were carried out. The maturity status had a greater effect on several anthropometric characteristics and on 15 m sprint, while it affected the CMJ only in U12 (*F* = 6.187, *p* ≤ 0.01). Differently, the RAE seemed to priorly affect the U13 and U15 categories in body composition, whereas its effect appeared on the 15-m sprint (*F*_(3, 45)_ = 4.147, *p* ≤ 0.01) and the RSA (*F*_(3, 45)_ = 3.179, *p* ≤ 0.05) in the U14 category. In addition, early matured players or those who were born in the first six months presented cellular characteristics similar to adult elite players. Soccer professionals should be encouraged to monitor the maturity status to better interpret changes in the physical performance of young soccer players to guide adequate training plans.

## 1. Introduction

Soccer is practiced all over the world, and male soccer players are among the most studied groups of athletes in sports sciences [1,2,3]. Soccer players at a high level require highly developed physical capacities, psychological factors, and perceptual, cognitive, and motor skills such as running, jumping, heading, kicking, passing, dribbling, and balance [4,5,6]. To achieve these goals, soccer academies play a fundamental role, as they guide the long-term development of young soccer players, with the main goal of further developing their skills and competencies. Youth selections are made early, to identify and develop talented individuals to compete at senior levels [7]. The possibility of joining high-level teams is an important opportunity for developing promising players [8], which is demonstrated by the significant differences between the elite and non-elite players in the youth category in physical and physiological characteristics [9].

Among the factors affecting both players’ selection and performance in youth soccer players, two of them play a fundamental role: the relative age effect (RAE) and biological maturation.

RAE refers to the asymmetric distribution of dates of birth in favor of players born at the beginning of the reference year concerning peers born at the end of the same year; players within the same age group can be by almost twelve months apart in chronological age. RAE has been demonstrated within different elite youth soccer academies [10,11,12,13]. Many studies affirm that a relatively greater age represents a performance advantage in experience and major physical, neural, motor and/or psychosocial maturity [10,14,15,16]. Therefore, there is an over-selection of relatively older players. These players are more likely to be identified as talented and recruited into academies and consequently provided with greater support and investment in their development [17].

Biological maturation can be defined as the timing and tempo of progress to achieving a mature state [18,19]. Maturity status is an important factor in the physical development of young players, especially concerning their body composition, physical capacities and match running performances [20,21,22]. Understanding the role of maturity on physical characteristics and performance in youth soccer players during adolescence is essential since this period coincides with the selection of players. Furthermore, it is important to consider that the physical demands of elite senior soccer players have increased rapidly in recent years, and this could cause recruiters and coaches to put greater emphasis on these aspects and physical fitness from an early age [23].

As far as we know, the association between RAE and maturation and their relationship with anthropometric characteristics, body composition and physical performance during adolescence, when players are selected at various competitive levels, have not been fully evaluated. Differences in maturity status and relative age have been identified in previous investigations, along with a considerable variation in timing and rate for physical and biological maturation [24,25]. Recent research reported that RAE and maturity status-related selection biases are separate processes and as such should be considered independently, but concluded that further research is required to better understand the nature and sources of the selection biases and how they may be used to optimize the opportunity for all youth players [10].

Thus, it is important to have an updated picture of how RAE and biological maturity affect the choices of players in teams, considering also what happens in teams of different levels, and how these two aspects affect the differences in anthropometric characteristics, body composition and physical performance of the players.

Therefore, the aim of this study was to evaluate the differences in RAE and biological maturity among the players of two Italian youth teams of different competitive levels, one elite and one non-elite and to understand the interaction effects amongst maturation status, and birth quartiles on physical characteristics and physical abilities of the players of the two groups. We assume that we will find a selection bias towards players who are born earlier and who are in an advanced maturity status in elite level players than their lower-level peers. In addition, we hypothesize that they also exhibit superior physical characteristics such as body composition and performance.

## 2. Materials and Methods

The below-mentioned research materials and methods are in line with our previous study that investigated different features in the same sample [26]. Several analyses are consequences of previous results.

### 2.1. Participants and Study Design

The design of the presented study was a cross-sectional experiment. The players of the two teams were examined on two different days in December 2021, from 10 a.m. to 6 p.m. Before enrolling the participants in the study, all the adolescents and their parents were informed about the experimental procedures and risks, and they could voluntarily decide to participate in the study. Although 191 samples were firstly enrolled, only 162 participants (13.01 ± 1.15 years) completed all the evaluations. No randomization was adopted. The Bologna (elite) Football Club 1990^®^ registered 98 attending soccer players who were divided in four categories (U12 = 18; U13 = 27; U14 = 30; U15 = 23), while the Russi (non-elite) Sports Union 1925^®^ registered 64 players (U12 = 16; U13 = 12; U14 = 21; U15 = 15).

The researchers did not collect information on diet attitudes. Also, no further information than the hours and frequency of training were collected. The Bologna’s young players trained four times per week for a total of six hours, whereas the Russi’s players trained three times per week for a total of four and a half hours.

Written informed consent was provided by the parents before the study began. The study was approved by the Bioethics Committee of the University of Bologna (Approval code: 25027).

### 2.2. Anthropometry

Three trained researchers cooperated and assessed the anthropometric evaluations according to standardized procedures [27]. Height and sitting height were measured to the nearest 0.1 cm using a stadiometer (GPM, Zurich, Switzerland), and leg length was derived by the subtraction of sitting height from height. Body weight was measured to the nearest 0.1 kg (light indoor clothing, without shoes) using a calibrated analogue scale. Circumferences (relaxed and contracted upper arm, thigh, and calf) were measured to the nearest 0.1 cm with a non-stretchable tape and widths (humerus and femur) to the nearest 0.1 cm with a sliding caliper, both on the left side of the body. The upper arm circumference was taken at the mid-point between the shoulder acromion and the olecranon process point, with the participant’s elbow relaxed along the body side (relaxed evaluation) or to be flexed 90° with palm facing upward (contracted evaluation); the thigh circumference was taken at the mid-point between the inguinal fold and the superior rotula point, with the participant in a standing position (thigh muscles relaxed); the calf circumference was taken at the bulkiest calf point, with the participant in a standing position (calf muscles relaxed); the humerus and femoral widths were taken, respectively, between the own lateral and medial epicondyles, with participants elbow and knee flexed 90°. Skinfold thicknesses (biceps, triceps, subscapular, supraspinal, sovrailiac, thigh, and calf) were measured to the nearest 1 mm using a Lange skinfold caliper at the left side of the body (Beta Technology Inc., Houston, TX, USA) at the following sites [28]: triceps and biceps, vertically at the mid-point between the acromion process and the olecranon process, respectively, at the posterior and anterior upper arm face; subscapular, at an angle of 45‣‣ to the lateral side of the body, about 20 mm below the tip of the scapula; sovrailiac, about 20 mm above the iliac crest (in the axillary line); thigh, vertically at the mid-point between the inguinal fold and the superior rotula point; calf, vertically at the bulkiest calf point both medially and laterally.

Finally, many measures were derived as in the previous literature. Body mass index (BMI) was computed as the ratio between the body weight (kg) and the stature squared (m^2^). Several parameters were estimated according to previous indications [29]. Four measures for the upper body and eight for the lower body were calculated: the total upper arm area (TUA, cm^2^), the upper mass area (UMA, cm^2^), the upper-fat area (UFA, cm^2^) and the upper-fat index (UFI, %); the total calf area (TCA, cm^2^), the calf mass area (CMA, cm^2^), the calf fat area (CFA, cm^2^), and the calf fat index (CFI, %); the total thigh area, (TTA, cm^2^), the thigh mass area (TMA, cm^2^), the thigh fat area (TFA, cm^2^), and the thigh fat index (TFI, %). Also, to calculate the body composition of each player, the skinfold equations developed by Slaughter and colleagues (1988) were used and three measures were gathered: the fat mass (FM, kg), the fat-free mass (FFM, kg), and the percentage of fat mass (%F, %).

### 2.3. Bioelectric Impedance Vector Analysis

A trained researcher performed the bioimpedance analysis using the BIA 101 anniversary analyzer (Akern^®^, Florence, Italy). The current frequency was settled at 50 kHz. A total body patient cable with four insulated alligator clips was used for connection to proximal (black) and distal (red) electrodes (Biatrodes^TM^, Florence, Italy). At the beginning of the evaluation day, the analyzer was tested to check the validity. To assess the evaluation, each participant was asked to lie down on a bed in the supine position, with a lower limb angle of 45° compared to the median line of the body and the upper limb angle of 30° from the trunk. Before recording the measurement, each participant waited two minutes to allow uniform distribution of bodily fluids. After cleansing the skin with alcohol, the electrodes (Ag/AgCl) were placed homolaterally on the right hand and foot, keeping them at least 5 cm apart [30].

The day before the evaluation, each participant was asked to abstain from foods and liquids for at least four hours before the test.

Vector length (VL) was calculated as (adjusted *R*^2^ + adjusted *Xc*^2^) 0.5 and PA as

$$arctg\frac{Xc}{R}\times \frac{180{}^{\circ}}{\pi }$$

BIVA was carried out using the classic methods, e.g., normalizing *R* (Ω) and *Xc* (Ω) for height in meters [31]. Elite male soccer players’ bioelectrical specific values [32] were used as a reference to build the 50%, 75%, and 95% tolerance ellipses on the *R*–*Xc* graph.

BIVA plots the parameters recorded in BIA (*R*, *Xc*, PhA) as a vector within a specific tolerance ellipses (specific profile for each sport and competitive level), and it allows to evaluate soft tissues through patterns based on percentiles of their electrical characteristics [33]. A BIVA vector that falls out of the 75% tolerance ellipses exhibits an abnormal tissue impedance, while vectors that fall in of the 50% represent a normal tissue impedance. BIVA outcomes could be interpreted by the vector direction to *x* and *y*-axis: vertical displacements indicate changes in tissue hydration (dehydration with long vectors, out of the upper pole; hyperhydration with short vectors, out of the lower pole); horizontal displacements indicate changes in soft tissue mass (more soft tissue to the left pole; less soft tissue to the right pole) [30].

### 2.4. Maturity Status

An estimation of the years from peak height velocity (PHV), which is an indicator for the adolescent growth spurt, was made using the equation for boys developed by Mirwald and colleagues [34].

Maturity offset = −9.236 + 0.0002708 (leg length ∗ sitting height) − 0.001663 (age ∗ leg length) + 0.007216 (age ∗ sitting height) + 0.02292 (weight/height).

Since maturity offset represents the time before or after PHV, the years from PHV were calculated by subtracting the age at PHV from chronological age.

In 2014, Malina and Koziel [35] reported that the approximation of the age at PHV (APHV), based on the prediction equation used, is often lower in younger children who are not yet in their adolescent growth spurt, and higher in older and sexually mature participants who already passed their adolescent growth spurt. To overcome this potential age effect, we followed the approach proposed by Rommers and colleagues [6], who used age-specific z-scores to classify players according to their maturity status. The predicted APHV was used to calculate z-scores within each specific age category (U10–U15, N = 6). Based on these age-specific z-scores of the predicted APHV, players were then classified as “earlier” (z < −0.5), “on-time” (−0.5 ≤ z ≤ 0.5), or “later” (z > 0.5) maturing [10,36].

### 2.5. Relative Age Effect (RAE)

Relative age was established from the birth date of each player and the cut-off date for the respective year group (1 January). As such, January was selected as the first month of the selection year and December was the last. The birth month of each player was compiled to define the birth quarter (Q), and four birth quartiles were designated: Q1 = January to March; Q2 = April to June; Q3 = July to September; Q4 = October to December.

### 2.6. Motor Tests

The performance tests were implemented at the University sports center. All participants performed three motor tests: the countermovement jump (CMJ), the 15 m straight-line sprint, and the repeated sprint ability (RSA). In addition, the soccer players who were 13 or more performed the Yo-Yo intermittent recovery test [26]. All the tests were preceded by a supervised and standardized warm-up consisting of 10 min of jogging, 5 min of athletic drills including jumping jack, lateral skip, high knee walk and backwards run, and 10 min of dynamic stretching of the lower limbs. A rest period of at least 3 min was allowed between different trials. Two electric photocells estimated the distance from the field through the jump duration during the CMJ test (OptoJump^®^, Microgate, Mahopac, New York, NY, USA). Also, a photoelectric cell timing system (Fusion Sport Smart Speed Timing Gates, Brisbane, Australia) estimated the time and distance covered during the 15 m sprint, RSA, and Yo-Yo tests.

The CMJ was assessed according to previous authors [37]. Before the evaluation, each participant was instructed to start from an upright position, making a rapid downward movement to a knee angle of 90° and simultaneously beginning to push off. The foot position coincided with the fitted acromion vertical line, with an extra-rotation at most of 15°. The hands were maintained on the waist for the entire trial. One minute of rest was allowed between the two attempts and the higher value was gathered.

The time to cover 15 m was detected on a football field and all participants wore technical clothes [38]. Players were positioned behind the start line (0.5 m) and were instructed to perform the sprint with maximal effort, after a sound start signal. Two trained coaches recorded the time to complete 15 m. Each athlete performed two attempts and the mean result was gathered.

The repeated sprint ability (RSA) consisted of six shuttle sprints of 40 m (20 + 20 m) with one change of direction (180°), as previously described [39]. Each shuttle was separated by 20 s of rest, after which the soccer player sprinted for 20 m, touched a line with a foot and came back to the starting line as fast as possible. One trial was assessed for each player and the best time (BT) in a single trial was measured and reported.

The Yo-Yo intermittent recovery test consisted of repeated 20 m runs back and forth between the starting, turning, and finish lines at a progressively increased speed, which is controlled by audio beeps from a tape recorder. When the participants failed twice to reach the finish line in time, the distance covered was recorded as the test result. This test consists of 4 running bouts at 10–13 km·h^−1^ and another 7 runs at 13.5–14 km·h^−1^, and then continues with stepwise 0.5 km·h^−1^ speed increments after every 8 running bouts (i.e., after 760, 1080, 1400, 1720 m, etc.) until exhaustion [40]. One trial was assessed for each player.

### 2.7. Statistical Analysis

The descriptive statistic was calculated and reported as mean ± standard deviation (SD) for continuous variables, while the frequency of appearance (percentage, %) was determined for qualitative variables (RAE and maturity status). The variables’ distribution was previously checked through graphics such as scatter plots, histograms, and box plots, and then verified with the Shapiro-Wilk test. When a variable showed a non-well-shaped distribution, a check for curve skewness and kurtosis was assessed. When the curve functions appeared right skewed, a location and scale (logarithm) transformation was applied.

The inference statistic was performed. Differences in frequencies were tested by the chi-squared (χ^2^) test and the Z test of proportion. In addition, the Risk Ratio (RR) was assessed and reported.

The two-way ANOVA was performed to compare differences between elite and non-elite players’ categories among RAE groups, and between elite and non-elite players’ categories among maturity status groups. A *p*-value (*p*) < 0.05 was considered significant. In addition, when an *F* value was significant, a post hoc Tukey evaluation was assessed to investigate among categories. However, only the *F* value (with its degrees of freedom) and the *p*-value (*p*) were reported.

## 3. Results

**Figure 1:**
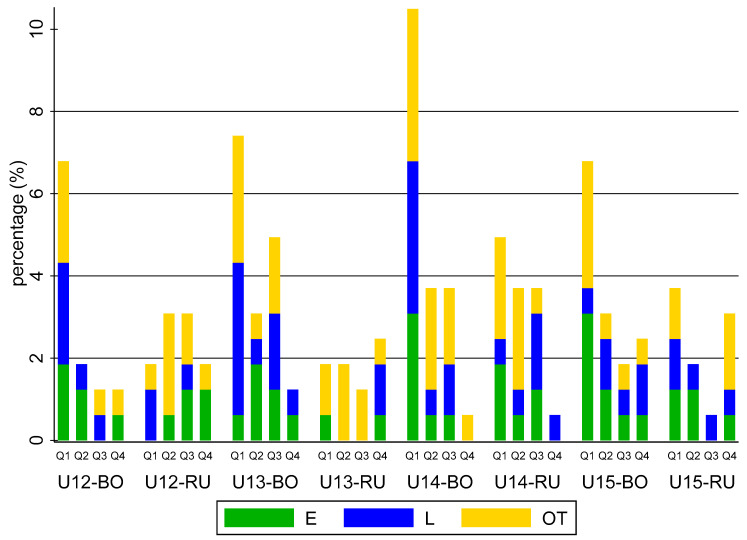
Percentage of soccer players for each team’s category over the maturity status and RAE.

Table 1 shows the prevalence differences in maturity status and RAE between Bologna F. C. and Russi U. S. among each category. The comparisons in maturity status between the two teams did not report significant differences, while the number of Bologna’s youngest players who were born between January and March was greater than those of Russi players. Despite several significant outcomes not arising, Figure 1 shows that the percentage of Bologna players who belonged to the first quartile (Q1: *n* = 51, 52.04%) was higher than that of other quartiles in each Bologna’s category (Q2: *n* = 19, 19.39%; Q3: *n* = 19, 19.39%; Q4: *n* = 9, 9.21%), and than the Q1 of Russi players (*n* = 18, 28.12%; RR = 1.85). However, the most of Russi players also belonged to first quartile (Q2: *n* = 17, 26.56%; Q3: *n* = 14, 21.87%; Q4: *n* = 13, 20.31%).

### 3.1. Maturity Status (MS)

Generally, significant differences resulted (Tables S1–S4) among each category for height and trunk height. The maturity status effect was greater with aging (Tables S2–S4), especially for weight, leg length, relaxed arm circumferences, humeral diameter, femoral diameter, total upper-body area, upper-body mass area, fat free mass. The maturity status had significant effects on 15-m sprint in U12, U13 and U14, while it affected the CMJ only in U12. Few measures were not affected by the maturity status in all categories such as subscapular, suprailiac and thigh skinfolds, calf, and thigh fat indexes, and the Yo-Yo IRT (U14 and U15, Tables S3 and S4).

With regard to the interaction between the maturity status and team membership (Tables S1–S4), it was significant in several measurements among the U13 category trunk height, BMI, relaxed arm circumference, thigh circumference, femoral diameter, subscapular skinfold, total upper-body area, upper-body mass area for U13, upper-body fat index, calf fat area, total thigh area for U13, thigh mass area, fat percentage, and fat mass. No significant results emerged among the other categories.

**Figure 2:**
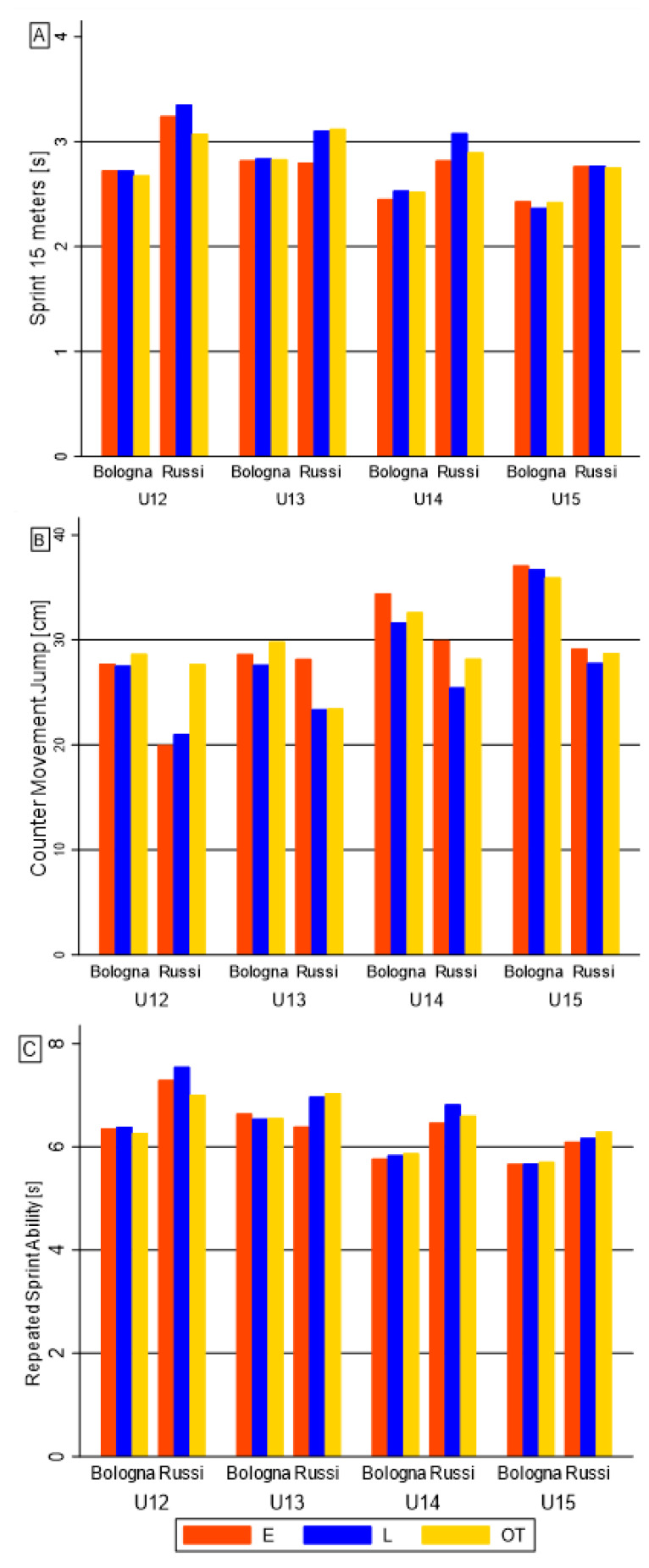
Bar graph of the maturity status effect on performance competition level discriminants, among teams and categories: (**A**) 15-meters sprint; (**B**) CMJ; (**C**) RSA.

**Figure 3:**
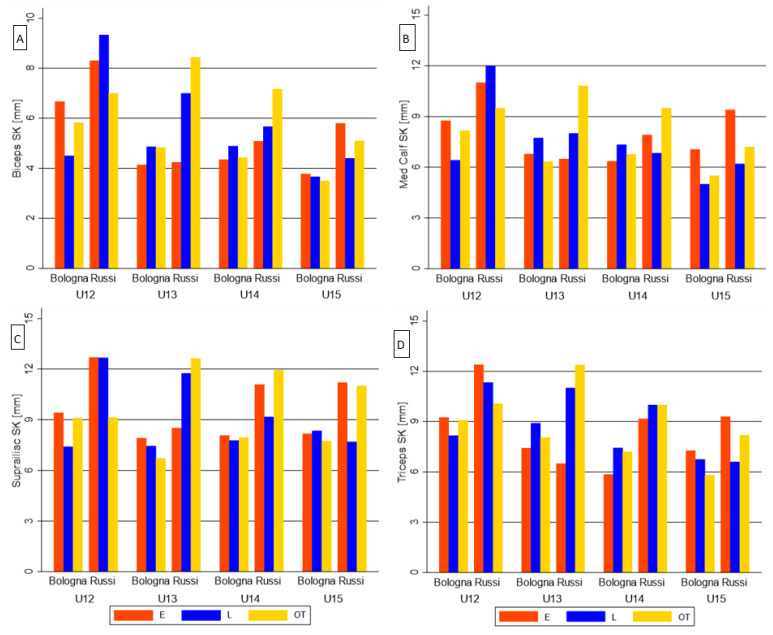
Bar graph of the maturity status effect on anthropometric competition level discriminants, among teams and categories: (**A**) Biceps SK; (**B**) Suprailiac SK; (**C**) Medial Calf SK; (**D**) Triceps SK.

Figure 2 and Figure 3 show the interaction between the maturity status, team membership and categories on better physical performance and anthropometric competition level discriminants, respectively. Regarding physical performance, the interaction differences between Bologna F.C. and Russi U.S. soccer players were significant on 15-m sprint in U13, on CMJ test in U12 category, and on RSA in U13 (Figure 2). Concerning body composition, the interaction comparisons resulted significant only on the medial calf skinfold in U13 (Figure 3).

### 3.2. Relative Age Effect (RAE)

Generally, no significant differences emerged from RAE comparisons (Tables S1–S4) for any parameters in all teams’ categories simultaneously. Also, the youngest group (Table S2) did not report significant outcomes between RAE quartiles. Differently, many significant differences appeared in U13 and U15 categories (Tables S2 and S4) for relaxed arm circumference, thigh circumference, biceps SK, supraspinal SK, thigh SK, total upper area, upper mass area, total thigh area, total mass, total fat area, total fat index, fat mass. Differences in U14 category were found in a few measurements: the fat free mass, the 15-m sprint, and the RSA.

**Figure 4:**
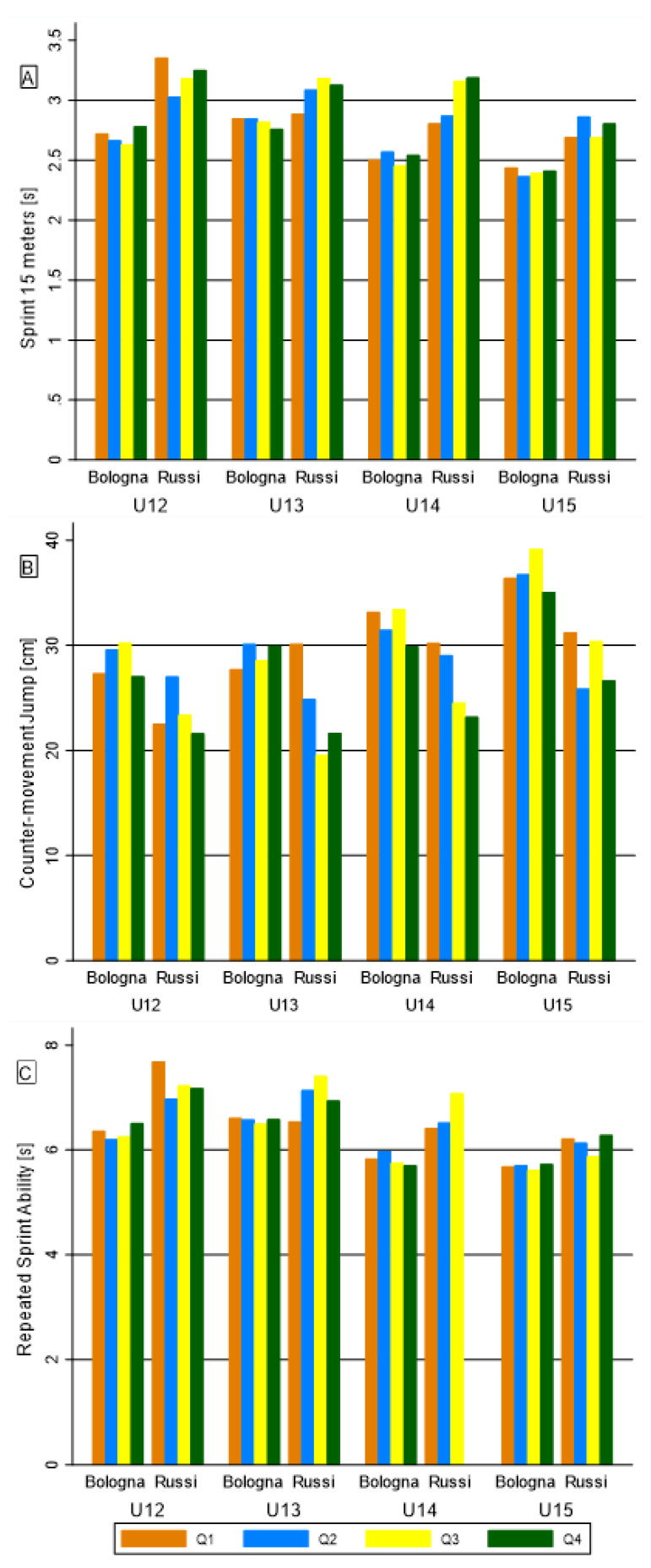
Bar graph of the Relative Age Effect on performance competition level discriminants, among teams and categories: (**A**) 15-m sprint; (**B**) CMJ; (**C**) RSA.

**Figure 5:**
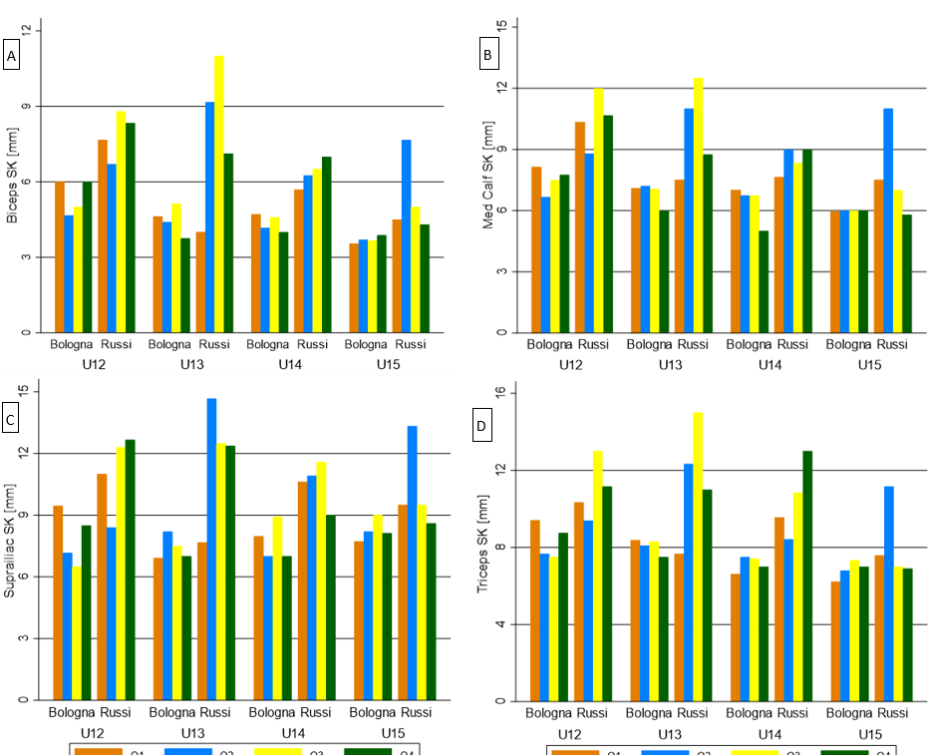
Bar graph of the Relative Age Effect on anthropometric competition level discriminants, among teams and categories: (**A**) Biceps SK; (**B**) Suprailiac SK; (**C**) Medial Calf SK; (**D**) Triceps SK.

Regarding the interaction between RAE and team membership (Tables S1–S4), few significant differences appeared in U13 and U15 categories for thigh SK and total fat area. A significant difference resulted in U14 on YO-YO IRT. In addition, Figure 4 and Figure 5 show the interaction between the RAE, team membership and categories on seven variables that previously discriminated among team levels [26]. In relation to physical performance (Figure 4), the interaction significantly differed for CMJ in U13 and for RSA in U13 and U14. In relation to body composition (Figure 5), the interaction comparisons resulted significant for biceps SK in U13 and U15, and for triceps SK in U13.

### 3.3. Bioimpedance Vector Analysis (BIVA)

**Figure 6:**
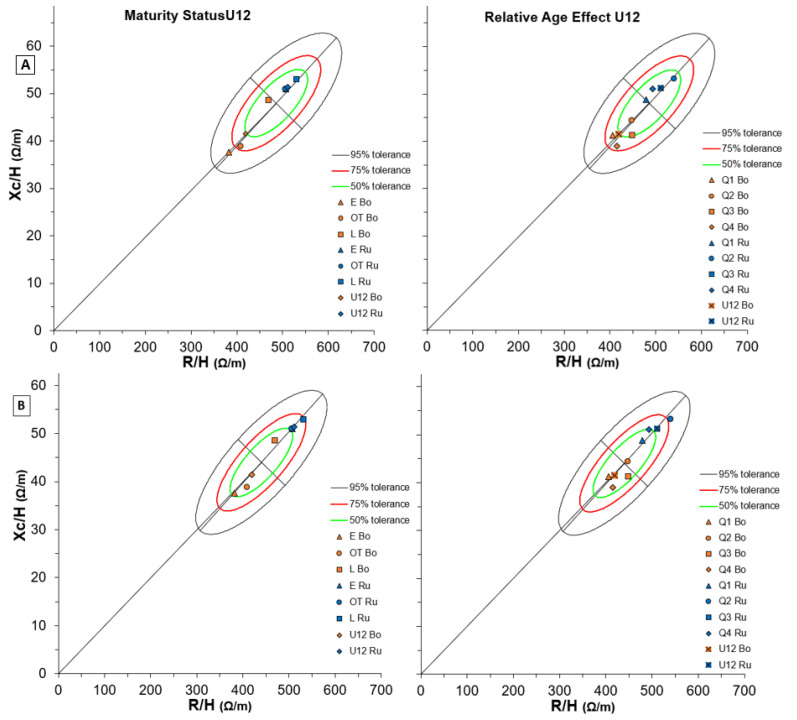
BIVA tolerance with Maturity Status (left) and Relative Age Effect (right) of both Bologna and Russi U12 groups for two reference populations: (**A**) number 112 (males, white, age 10–11 years, BMI 18, Italy, Akern-RJL Systems); (**B**) number 114 (males, white, age 12 years, BMI 18, Italy, Akern-RJL Systems). Note: U15 Bo and U15 Ru refer to team means respectively.

Figure 6 shows BIVA results in U12 soccer players of both teams for Maturity Status (left side) and RAE (right side) considering two different reference populations (A and B). Generally, elite U12 players reported greater cellularity than non-elite. As regards maturity status, several differences appeared within and between teams’ comparisons. Firstly, the 12-years-old white-male ellipse appeared the most adequate for the elite team (Figure 6B, left side), while the non-elite team fell better in the 10–11 years-old white-male ellipse (Figure 6A, left side). In addition, when compared to the Serie A soccer players graph (Figure S1), Bologna soccer players, who matured earlier or on time, were the closest to the ellipse, while the later matured players were the farthest.

Regarding RAE, players who were in quartile 1 of both teams showed cellularity more similar to elder reference populations (Figure S1), but this trend was not linear with the increasing quartiles.

**Figure 7:**
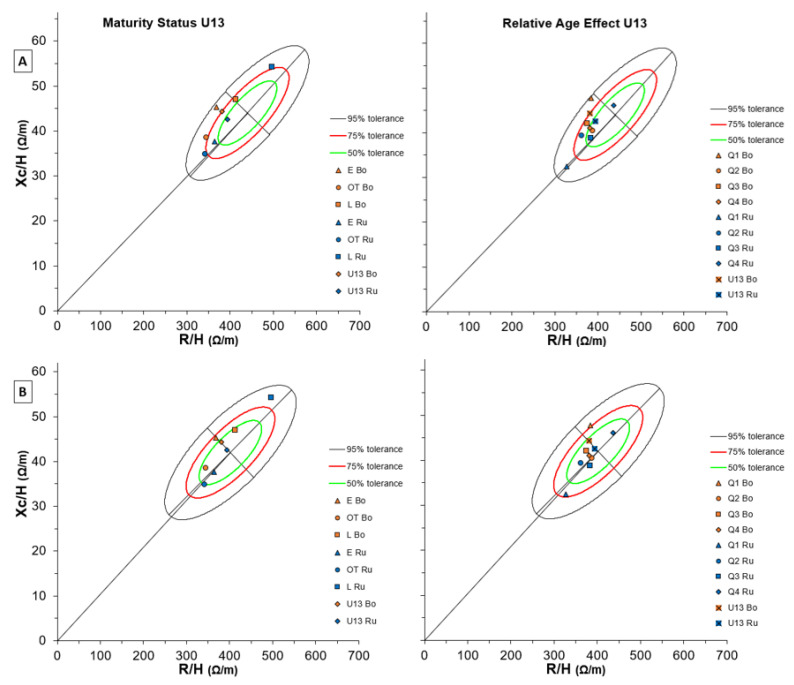
BIVA tolerance with Maturity Status (left) and Relative Age Effect (right) of both Bologna and Russi U13 groups for two reference populations: (**A**) number 114 (males, white, age 12 years, BMI 18, Italy, Akern-RJL Systems); (**B**) number 116 (Males + Females, White, age 13 years, BMI 19, Italy, Akern-RJL Systems). Note: U15 Bo and U15 Ru refer to team means, respectively.

Figure 7 shows BIVA results in U13 soccer players of both teams for Maturity Status (left side) and RAE (right side) considering two different reference populations (A and B). Generally, the means of the two teams presented close positions in the graph and the 13 years-old white-male reference ellipse appeared the most adequate for both elite and non-elite players. As regards maturity status, earlier players’ cells’ characteristics resulted closer to the Serie A soccer players’ ellipse (Figure S2). In contrast, players who matured later appeared farthest from the elite men’s graph, especially in Russi U.S. (Figure S2).

Regarding RAE, despite players who were born in the first quartile laid on a 75% tolerance line, the graph and the 13 years-old white-male reference ellipse appeared the most adequate for both teams (Figure 7B, right side). However, the earlier non-elite team players were the closest to the Serie A soccer players’ ellipse, while the elite team players did not result affected by the RAE and showed similar cell characteristics among quartiles (Figure S2).

**Figure 8:**
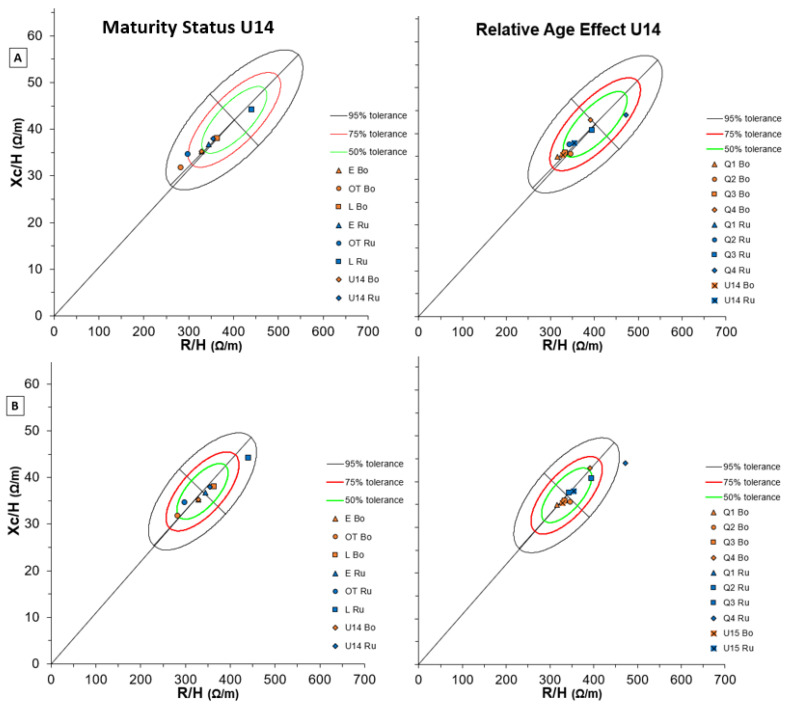
BIVA tolerance with Maturity Status (left) and Relative Age Effect (right) of both Bologna and Russi U14 groups for two reference populations: (**A**) number 116 (males, white, age 13 years, BMI 19, Italy, Akern-RJL Systems); (**B**) number 118 (Males + Females, White, age 14–15 years, BMI 20, Italy, Akern-RJL Systems). Note: U15 Bo and U15 Ru refer to team means respectively.

Figure 8 shows BIVA results in U14 soccer players of both teams for Maturity Status (left side) and RAE (right side) considering two different reference populations (A and B). Generally, most of the means of the two teams lay on the 50% tolerance line in the 14–15 years-old white-male reference ellipse (Figure 8B). As regards maturity status, earlier players of the two teams showed more similar characteristics to Serie A adult players (Figure S3), while the latter players moved up and to the right on the 14–15 years ellipses (Figure 8B).

Regarding RAE, the elder players (Q1 and Q2) of both teams showed similar characteristics and were nearer to elite adult players (Figure S3). However, the 14–15 years-old white-male reference ellipse better described the body composition of the elder U14 soccer players (Figure 8B), while the younger (Q3 and Q4) better laid in the 13 years-old white-male reference graph (Figure 8A).

**Figure 9:**
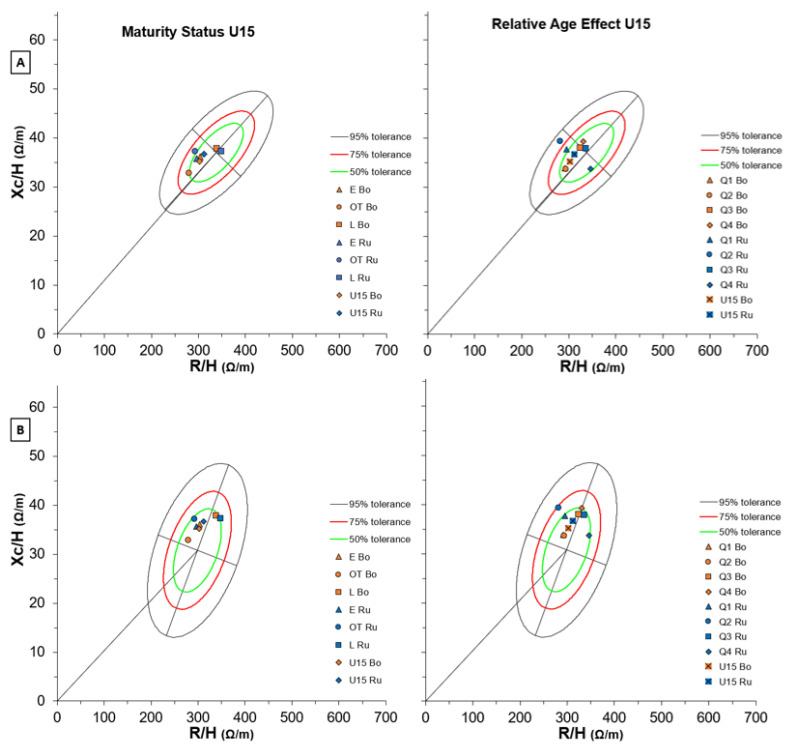
BIVA tolerance with Maturity Status (left) and Relative Age Effect (right) of both Bologna and Russi U14 groups for two reference populations: (**A**) number 118 (Males + Females, White, age 14–15 years, BMI 20, Italy, Akern-RJL Systems); (**B**) the number 1 (Males, White, 16 age 85 years, 16 BMI 31, Italy, Akern-RJL Systems). Note: U15 Bo and U15 Ru refer to team means respectively.

Figure 9 shows BIVA results in U15 soccer players of both teams for Maturity Status (left side) and RAE (right side) considering two different reference populations (A and B). As regards maturity status, the earlier players were better described by the 16–85 year-old white-male reference ellipse (Figure 9B), while the latter players appeared similar to the 14–15 years-old white-male population (Figure 9A). Also, the earlier players’ cells were more similar to Serie A men than the latter U15 players (Figure S4).

Regarding RAE, the elder players (Q1 and Q2) showed more athletic characteristics than their younger teammates (Figure 9B), who appeared similar to the 14–15 years-old white-male population (Figure 9A). However, non-elite team players who were born between January and June lay in the 95% tolerance line of Serie A adult players (Figure S4).

## 4. Discussion

The main aim of this study was to evaluate the differences in maturity status and relative age effect among the players of two Italian youth teams of different competitive levels, one elite and one non-elite. We found that the two teams did not show significant differences in the frequencies of maturity status, while few differences in RAE emerged. The percentage of the Bologna players who belonged to the first quartile was higher than those observed for Russi players in all age groups. Thus, the overall RAE for the elite soccer players showed that players born at the beginning of the year were consistently over-represented. These results are in line with those reported in several elite soccer leagues worldwide [41,42,43,44,45,46,47,48,49,50,51,52,53,54]. In addition, the results confirmed that RAE was more prevalent in the clubs and academies classified in the highest level of certification [44,55]. According to Figueiredo et al. [44], this might suggest that clubs and academies certified as training institutions also have the means to select more players than the lower-level certification clubs and academies, thus taking advantage of the potential beneficial effect of an over-representation of the chronologically older players. In our study, the prevalence of players born in Q1 was particularly evident in U14. Prior studies have reported that the extent of the RAE decreases with increasing age, with evidence after adolescence [14,41,56,57,58].

Regarding maturity status between the two competitive levels, we did not find differences in prevalence, despite other authors reporting differences among the competitive levels [24]. However, previous studies reported that the chance of selection for relatively younger soccer players is higher only if they were early maturing whereas relatively older athletes had a selection advantage independent of their maturity status [59,60].

The second aim was to understand the interaction effects amongst maturation status, and birth quartiles on the players’ physical characteristics and abilities. The results have shown that the maturity status had greater effects than RAE in both anthropometry and motor tests. The magnitude of the effects was seen to vary with age and the maturity status effect was bigger in U12 and U14 than in RAE.

The effects of RAE in the two teams were observed only in U13 and in U15, with significant differences for some anthropometric parameters (weight, circumferences, fat parameters and many of the limb areas). In contrast, RAE was unrelated to performance tests and only significantly associated with superior sprint 15 m and RSA performance in players born in the first months of the years U14. The results follow what was reported by other authors [56,61]. Cobley et al. [56] in their meta-analysis showed a small-moderate effect for individuals aged 15–18 years that declined for older individuals, while Peña-González et al. [61] affirmed that anthropometrical and physical performance differences observed in different competitive levels are not due to the relative age but principally to the level of competition. In addition, some authors found that earlier birthdates (quartile one) were not associated with the likelihood to be selected or promoted to a higher level in soccer players [62].

The effects of the differences in biological maturity were evident for all the age groups, and regard both the anthropometric characteristics and performance tests, indicate that maturity has a greater association with physical characteristics and physical abilities than RAE in Italian male youth soccer players. The current study found that early mature subjects were taller, and heavier, and presented better body composition parameters and performance than youths who matured on time or late. Similar findings have been reported in other studies [7,24,55,63], where maturity status was shown to have a much greater influence on anthropometry and physical characteristics than RAE in young soccer players. Johnson et al. [24] reported that maturation status had an even 10-fold stronger influence on selection in elite youth soccer than the relative age.

Maturation affected physical performance, with early maturing boys performing better than them on time and late peers, and this had a subsequent impact on match performance in soccer [64]. However, it should be considered that, although advanced maturity offers an initial advantage in terms of performance and selection, in the long term this can be counterproductive [7]. Players who mature early tend to overlook their technical and tactical development in favor of the use of their physical ability [65]. In elite soccer, there is the gradual exclusion of early matured players and the selection of those who matured late with increasing age [7]. Caution must be taken in assessing relationships between RAE, maturation, and performance. Physical advantages related to age and/or maturation during adolescence are highly transitory and tend to disappear or even reverse in adulthood. Those involved in the identification and in the development of the academy players should be aware of and accommodate for individual differences in maturation.

The last purpose of this study was to evaluate the biological maturity and the relative age effect on bioimpedance parameters. To interpret the BIVA outcomes well, one of the most relevant features is to compare the analyzed sample to an adequate reference population. In adolescent players, the faster change of maturity stages requests rigorous analysis. We found that players who matured earlier had similar cellularity to elder adolescent and adult players, independently of team level. The effect of the elite team was more evident in U12 and U15 soccer players. Although previous studies follow biological maturity influence, this effect seems to be more pronounced in soccer players’ body fluids [22,66]. However, to the best of our knowledge, no authors investigated the biological maturity effect on BIVA at two competitive soccer team levels using different reference population graphs.

Regarding RAE, despite the elder reference population ellipse including most of the observations in the 50% tolerance line, the quartiles showed different trends among the categories. Players who were born in the first six months of the year exhibited greater cellularity in U12, U14 and U15, while in U13 this discrepancy is evident only in non-elite team players. Also, elite players showed characteristics more similar to adult soccer players only in U12 and U14 categories. To the best of our knowledge, no authors investigated the RAE on BIVA in younger soccer players and more evidence is needed.

The results found are of great importance for coaches and other professionals responsible for the process of scouting and training young soccer players. These professionals should be aware of the different stages of growth and biological maturation and their influences on different body dimensions and performance. Following our results, relative age should be considered as a secondary factor in the process of identification, selection, and development of young soccer players.

This study presented many limitations: (1) maturity was not assessed using the gold standard method of skeletal maturity; (2) due to the presence of three or four groups for maturity status and RAE respectively, a bigger sample size should evidence many differences; (3) no specific soccer performance test was assessed.

In conclusion, maturity status and relative age were differentially associated with physical characteristics and physical abilities in young soccer players. Specifically, advanced maturity was associated with better anthropometric characteristics and superior performance in most age groups, whereas relative age was, in the majority of cases, unrelated to performance.

The findings from the current study expand on this previous research, identifying that maturity influences anthropometric characteristics and performance rather than RAE between 12 and 15 years.

## 5. Conclusions

The main purpose of the present study was to evaluate the differences in RAE and biological maturity among the players of two Italian youth teams of different competitive levels, one elite and one non-elite and to assess the relationship between maturation, age, and relative physical and performance characteristics. The characteristics analyzed are mainly associated with maturation, while the relationship with RAE is less evident. Professionals should understand that RAE and maturity status are two distinct constructs. Coaches and other professionals involved should be encouraged to monitor growth and maturation to better interpret changes in the physical performance of young soccer players. Maturity status should be taken into consideration both in making the selections, but also to guide training, and to mitigate the differences due to the different maturity statuses.

## Figures and Tables

**Table 1:** Analysis of maturity and RAE proportions among categories of each football team.

	∆ Bologna-Russi U12			∆ Bologna-Russi U13			∆ Bologna-Russi U14			∆ Bologna-Russi U15		
**Maturity**	Z or χ^2^	*p*	RR	Z or χ^2^	*p*	RR	Z or χ^2^	*p*	RR	Z or χ^2^	*p*	RR
E	0.232	0.817	1.067	1.065	0.287	1.556	−0.423	0.6725	0.817	0.359	0.717	1.174
L	0.752	0.452	1.778	1.630	0.103	2.444	0.11	0.912	1.050	−0.482	0.630	0.783
OT	−0.424	0.671	0.667	0.178	0.859	0.500	0.269	0.788	1.089	0.092	0.926	1.043
Total	1.263	0.532		3.871	0.144		0.181	0.913		0.2531	0.881	
**RAE**												
Q1	2.505	0.012 *	3.259	1.152	0.249	1.778	1.302	0.192	1.487	0.474	0.635	1.196
Q2	−1.007	0.317	0.533	−0.463	0.644	0.741	−0.710	0.478	0.7	0.128	0.898	1.087
Q3	−1.449	0.147	0.355	0.856	0.392	1.778	−0.710	0.478	0.7	0.626	0.531	1.956
Q4	−0.628	0.530	0.593	−2.071	0.038 *	0.222	−0.259	0.796	0.7	−1.129	0.258	0.5
Total	6.462	0.091		5.161	0.160		1.705	0.636		1.462	0.691	

*Note*: E, early; L, late; OT, on time; RAE, relative age effect; Q1, quartile 1; Q2, quartile 2; Q3, quartile 3; Q4, quartile 4; Z, the test of proportion Z; χ^2^, Pearson chi-squared test; *p*, *p*-value; RR, risk ratio; *, statistically significant; ∆, difference.

## Data Availability

Not applicable.

## Supplementary Materials

The following supporting information can be downloaded at: https://www.mdpi.com/article/10.3390/biology11111559/s1, Table S1: Variables mean comparisons and interaction effects of Maturity Status, Teams, and RAE in U12 soccer players; Table S2: Variables mean comparisons and interaction effects of Maturity Status, Teams, and RAE in U13 soccer players; Table S3: Variables mean comparisons and interaction effects of Maturity Status, Teams, and RAE in U14 soccer players; Table S4: Variables mean comparisons and interaction effects of Maturity Status, Teams, and RAE in U15 soccer players; Figure S1: BIVA tolerance with Maturity Status (top) and Relative Age Effect (bottom) of both Bologna and Russi U12 groups for Italian SERIE A reference populations (number 20, Serie A Micheli). Note: U15 Bo and U15 Ru refer to team means respectively; Figure S2: BIVA tolerance with Maturity Status (top) and Relative Age Effect (bottom) of both Bologna and Russi U13 groups for Italian SERIE A reference populations (number 20, Serie A Micheli). Note: U15 Bo and U15 Ru refer to team means respectively; Figure S3: BIVA tolerance with Maturity Status (top) and Relative Age Effect (bottom) of both Bologna and Russi U14 groups for Italian SERIE A reference populations (number 20, Serie A Micheli). Note: U15 Bo and U15 Ru refer to team means respectively; Figure S4: BIVA tolerance with Maturity Status (top) and Relative Age Effect (bottom) of both Bologna and Russi U15 groups for Italian SERIE A reference populations (number 20, Serie A Micheli). Note: U15 Bo and U15 Ru refer to team means respectively.
