# Supplementary material for: Maturation Selection Biases and Relative Age Effect in Italian Soccer Players of Different Levels
Source: Biology (Basel). 2022 Oct 24;11(11):1559. doi: 10.3390/biology11111559 (PMC9687510; doi:10.3390/biology11111559)
Supplement: Supplementary file 1 [file biology-11-01559-s001.zip › Figure S3.pdf]

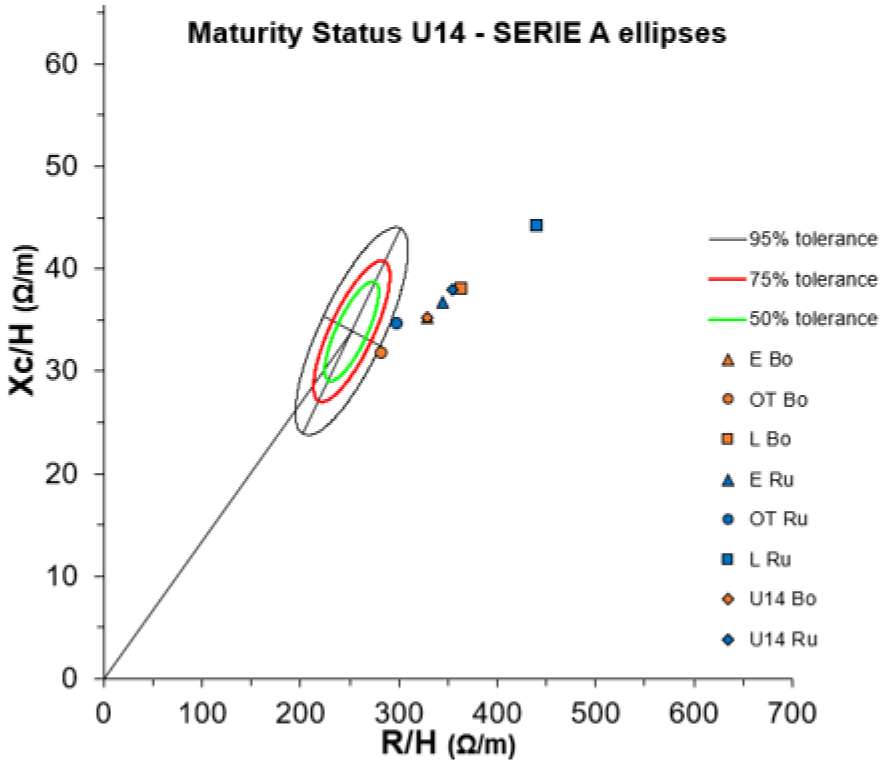

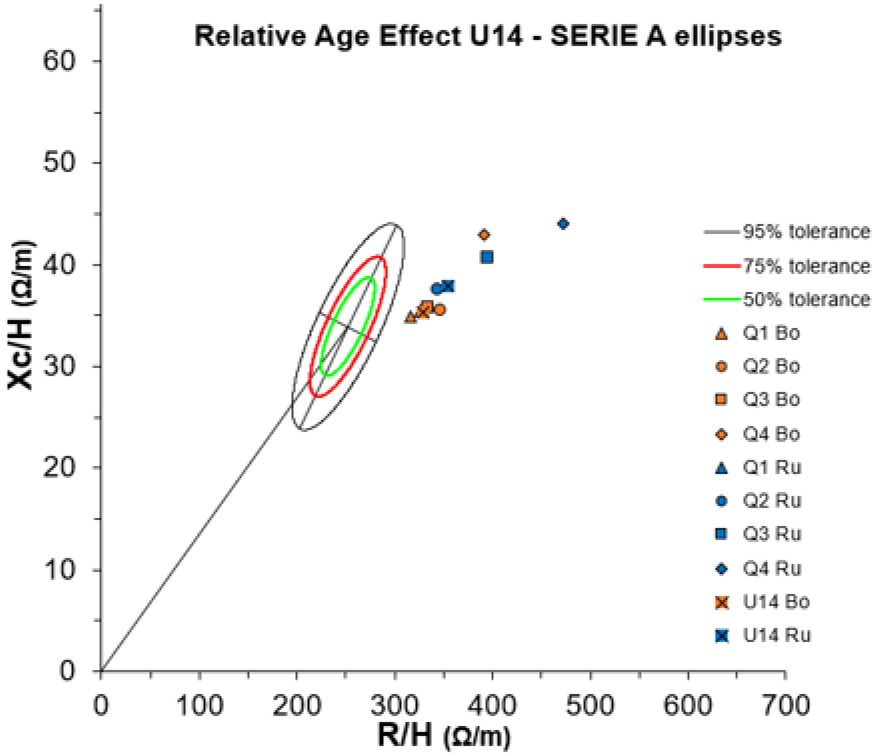

Figure S3. BIVA tolerance with Maturity Status (top) and Relative Age Effect (bottom) of both Bologna and Russi U14 groups for Italian SERIE A reference populations (number 20, Serie A Micheli). Note: U15 Bo and U15 Ru refer to team means respectively.
