# Supplementary material for: Maturation Selection Biases and Relative Age Effect in Italian Soccer Players of Different Levels
Source: Biology (Basel). 2022 Oct 24;11(11):1559. doi: 10.3390/biology11111559 (PMC9687510; doi:10.3390/biology11111559)
Supplement: Supplementary file 1 [file biology-11-01559-s001.zip › Table S1.pdf]

Table S1. Variables mean comparisons and interaction effects of Maturity Status, Teams and RAE in U12 soccer players.

	U12 Bologna			U12 Russi						U12 Bologna				U12 Russi						
	E (n=6)	OT (n=6)	L (n=6)	E (n=5)	OT (n=8)	L (n=3)	Bo-Ru	MS	teams*MS	Q1 (n=11)	Q2 (n=3)	Q3 (n=2)	Q4 (n=2)	Q1 (n=3)	Q2 (n=5)	Q3 (n=5)	Q4 (n=3)	Bo-Ru	RAE	teams*RAE
	Mean (± SD)	Mean (± SD)	Mean (± SD)	Mean (± SD)	Mean (± SD)	Mean (± SD)	F $_{(1, 31)}$	F $_{(2, 31)}$	F $_{(5, 28)}$	Mean (± SD)	Mean (± SD)	Mean (± SD)	Mean (± SD)	Mean (± SD)	Mean (± SD)	Mean (± SD)	Mean (± SD)	F $_{(1, 30)}$	F $_{(3, 30)}$	F $_{(7, 26)}$
Weight (Kg)	46.50 (4.51)	42.33 (5.65)	35.00 (6.00)	41.70 (6.61)	38.12 (7.46)	40.00 (15.13)	0.265	2.012	1.301	42.09 (5.56)	40.67 (8.50)	36.00 (12.73)	43.00 (12.73)	37.33 (10.69)	39 (5.80)	43.3 (9.98)	36.67 (9.71)	0.180	0.001	0.820
Height (cm)	159.03 (5.00)	154.30 (8.09)	145.20 (4.36)	144.30 (4.56)	141.82 (4.82)	141.30 (6.00)	27.032†	5.290‡	2.365	153.60 (7.65)	155.40 (8.21)	143.75 (8.84)	153.95 (12.23)	140.87 (5.32)	142.90 (5.72)	145.30 (1.64	138.80 (5.21)	13.117†	0.520	1.818
Trunk Height (cm)	80.82 (2.46)	78.10 (1.65)	73.27 (2.39)	74.74 (1.94)	72.57 (1.19)	66.17 (7.23)	41.929†	21.346†	0.218	77.28 (3.74)	78.23 (4.82)	74.35 (3.46)	79.80 (4.10)	71.23 (2.85)	73.46 (1.68)	70.46 (7.01)	73.17 (3.07)	10.368‡	1.078	0.128
Leg lenght (cm)	78.22 (3.90)	76.20 (6.69)	71.93 (4.38)	69.56 (3.55)	69.25 (4.21)	75.13 (12.44)	4.230*	0.139	2.928	76.32 (5.53)	77.17 (3.51)	69.40 (5.37)	74.15 (8.13)	69.63 (2.92)	69.44 (4.15)	74.84 (8.55)	65.63 (2.65)	3.904	0.422	2.022
BMI (kg/m²)	18.36 (1.10)	17.74 (1.55)	16.53 (2.19)	20.08 (3.52)	18.91 (3.48)	19.69 (5.72)	3.667	0.412	0.294	17.78 (1.39)	16.69 (1.86)	17.14 (4.04)	17.89 (2.52)	18.60 (3.91)	19.17 (3.43)	20.46 (4.42)	18.92 (4.49)	2.502	0.092	0.253
Relaxed arm circ. (cm)	21.73 (1.32)	21.72 (1.51)	19.20 (2.13)	22.62 (3.53)	21.56 (3.27)	22.37 (3.74)	1.880	0.643	1.002	21.55 (1.54)	19.87 (1.60)	19.40 (4.81)	20.20 (1.70)	21.70 (2.66)	21.36 (3.02)	23.40 (3.53)	21.27 (4.60)	2.374	0.241	0.591
Contracted arm circ. (cm)	23.22 (1.21)	22.82 (1.67)	20.68 (2.19)	24.02 (3.56)	22.82 (3.25)	23.67 (3.81)	1.750	0.696	0.798	22.81 (1.51)	21.80 (1.91)	20.65 (4.74)	21.35 (2.05)	23.03 (2.80)	22.70 (3.28)	24.46 (3.66)	22.93 (4.30)	2.152	0.116	0.507
Calf circ. (cm)	32.77 (0.93)	30.90 (1.93)	28.05 (1.82)	32.00 (2.93)	30.66 (2.68)	30.93 (6.02)	0.434	2.830	1.241	30.66 (2.18)	30.77 (2.89)	29.80 (5.52)	30.55 (3.32)	29.60 (3.86)	31.14 (2.72)	32.46 (3.82)	30.43 (3.73)	0.143	0.160	0.433
Thigh circ. (cm)	42.70 (2.85)	41.58 (3.19)	38.28 (3.24)	44.88 (5.37)	44.09 (5.22)	42.87 (7.58)	3.674	1.214	0.189	41.85 (2.59)	38.60 (1.78)	39.60 (8.77)	40.05 (5.16)	42.37 (6.78)	44.08 (5.00)	45.50 (5.94)	43.57 (6.16)	4.236*	0.076	0.540
Humeral diameter (mm)	6.10 (0.18)	5.87 (0.46)	5.67 (0.39)	5.90 (0.54)	5.73 (0.33)	5.53 (0.49)	1.237	2.376	0.022	5.89 (0.39)	5.93 (0.55)	5.65 (0.49)	5.95 (0.21)	5.57 (0.55)	5.74 (0.43)	5.90 (0.48)	5.67 (0.29)	0.650	0.094	0.577
Femoral diameter (mm)	8.90 (0.33)	8.62 (0.40)	8.27 (0.40)	9.12 (0.54)	8.88 (0.57)	8.80 (0.75)	3.690	2.255	0.273	8.62 (0.35)	8.63 (0.40)	8.35 (0.92)	8.65 (0.92)	8.83 (0.80)	9.12 (0.47)	8.88 (0.53)	8.83 (0.81)	2.686	0.256	0.175
Triceps SK (mm)	9.25 (2.68)	9.08 (2.65)	8.17 (3.30)	12.40 (3.29)	10.06 (3.05)	11.33 (2.52)	5.220*	0.590	0.535	9.41 (2.70)	7.67 (1.53)	7.50 (3.54)	8.75 (5.30)	10.33 (1.15)	9.40 (2.07)	13.00 (2.92)	11.17 (5.01)	5.252*	0.478	0.768
Biceps SK (mm)	6.67 (1.89)	5.83 (2.07)	4.50 (1.84)	8.30 (2.99)	7.00 (3.12)	9.33 (3.21)	7.797‡	0.536	1.426	6.00 (2.05)	4.67 (0.58)	5.00 (2.12)	6.00 (4.24)	7.67 (0.58)	6.70 (3.73)	8.80 (3.03)	8.33 (4.04)	5.281*	0.402	0.195

Subscapular SK (mm)	6.42 (1.91)	6.50 (1.64)	5.00 (1.26)	8.50 (3.91)	6.81 (4.29)	6.83 (1.61)	1.841	0.665	0.333	6.23 (1.37)	5.00 (1.00)	5.00 (1.41)	7.00 (4.24)	7.167 (1.89)	7.00 (5.05)	7.00 (2.67)	8.67 (5.51)	1.906	0.439	0.057
Supraspinal SK (mm)	6.42 (2.01)	7.17 (2.32)	5.42 (2.08)	9.20 (4.66)	7.06 (4.14)	9.00 (3.46)	3.168	0.150	1.002	6.82 (1.85)	5.17 (2.02)	5.75 (3.18)	6.00 (4.24)	7.67 (1.15)	6.30 (4.62)	9.40 (3.78)	9.33 (6.03)	2.910	0.499	0.317
Suprailiac SK (mm)	9.42 (2.91)	9.08 (3.14)	7.42 (3.23)	12.70 (5,45)	9.13 (4.36)	12.67 (4.04)	4.153	0.760	1.225	9.45 (2.83)	7.17 (0.76)	6.50 (3.54)	8.50 (6.36)	11.00 (1.73)	8.40 (4.34)	12.30 (4.76)	12.67 (7.51)	4.046	0.676	0.484
Thigh SK (mm)	11.33 (1.89)	11.08 (2.46)	9.75 (3.00)	13.60 (3.65)	11.94 (3.49)	13.33 (5.13)	3.826	0.320	0.479	11.14 (2.58)	9.50 (0.87)	10.00 (2.83)	11.00 (4.24)	11.33 (2.08)	11.90 (4.10)	14.40 (4.10)	12.67 (4.16)	2.883	0.271	0.520
Medial Calf SK (mm)	8.75 (1.99)	8.17 (3.13)	6.42 (2.91)	11.00 (2.92)	9.50 (2.67)	12.00 (4.36)	8.721‡	0.399	1.417	8.14 (2.79)	6.67 (1.53)	7.50 (3.54)	7.75 (5.30)	10.33 (1.53)	8.80 (3.11)	12.00 (3.46)	10.67 (3.21)	6.117*	0.583	0.215
Lateral Calf SK (mm)	9.25 (1.17)	8.25 (3.06)	7.67 (3.08)	11.20 (3.29)	9.81 (2.64)	12.50 (3.97)	7.488‡	0.642	0.917	8.77 (2.52)	8.17 (1.26)	8.00 (4.24)	7.00 (4.24)	10.83 (1.26)	8.90 (3.05)	12.40 (3.60)	11.00 (3.00)	6.143*	0.472	0.576
TUA (cm^2)	37.72 (4.61)	37.70 (5.07)	29.65 (6.43)	41.53 (12.70)	37.76 (11.32)	40.57 (13.75)	2.240	0.557	0.884	37.16 (5.17)	31.56 (5.08)	30.89 (14.85)	32.60 (5.46)	37.87 (9.30)	36.90 (11.03)	44.39 (12.66)	37.13 (15.65)	2.566	0.241	0.539
UMA (cm^2)	30.47 (4.58)	30.41 (4.86)	23.06 (6.42)	35.11 (12.63)	30.92 (10.95)	33.85 (13.41)	2.712	0.540	0.826	30.04 (5.05)	24.48 (4.05)	24.10 (13.98)	25.81 (6.36)	30.97 (8.69)	29.87 (10.55)	37.93 (12.46)	30.85 (15.53)	2.983	0.268	0.579
UFA (cm^2)	7.25 (0.77)	7.29 (0.75)	6.59 (0.57)	6.42 (0.51)	6.84 (0.64)	6.72 (0.34)	2.776	1.091	1.307	7.13 (0.72)	7.08 (1.03)	6.79 (0.87)	6.79 (0.90)	6.90 (0.64)	7.03 (0.59)	6.46 (0.47)	6.28 (0.31)	1.080	0.948	0.118
UFI (%)	19.42 (2.81)	19.61 (3.25)	23.12 (5.16)	16.64 (5.03)	19.40 (5.31)	17.61 (4.66)	3.077	0.666	0.892	19.51 (3.33)	22.48 (0.43)	24.08 (8.76)	21.37 (6.35)	18.70 (3.04)	19.91 (3.83)	15.63 (4.89)	19.16 (8.47)	3.785	0.299	0.871
TCA (cm^2)	85.54 (4.85)	76.27 (9.48)	62.86 (8.14)	82.08 (14.81)	75.36 (13.37)	78.11 (30.76)	0.581	2.458	1.312	75.21 (10.52)	75.81 (13.86)	71.91 (26.17)	74.75 (16.17)	70.55 (18.34)	77.68 (13.86)	84.82 (20.07)	74.48 (18.31)	0.164	0.178	0.393
CMA (cm^2)	58.61 (4.75)	52.97 (7.23)	44.49 (3.08)	50.39 (11.07)	48.33 (5.83)	43.63 (12.98)	3.063	4.756‡	0.594	51.46 (8.14)	54.55 (9.45)	49.87 (12.20)	53.49 (1.35)	42.42 (11.04)	52.32 (7.43)	49.14 (6.62)	44.97 (12.08)	2.286	0.800	0.418
CFA (cm^2)	26.93 (4.45)	23.30 (8.85)	18.37 (8.25)	31.69 (9.58)	27.03 (9.18)	34.47 (17.90)	6.020‡	0.614	1.244	23.74 (7.48)	21.26 (5.24)	22.04 (13.97)	21.26 (14.82)	28.12 (7.30)	25.36 (10.35)	35.68 (13.65)	29.51 (9.56)	3.861	0.351	0.336
CFI (%)	31.44 (4.50)	30.07 (9.71)	28.34 (9.25)	38.54 (8.84)	35.16 (6.33)	42.83 (5.10)	10.462‡	0.480	0.983	31.23 (8.26)	27.87 (3.25)	29.04 (8.86)	26.93 (14.01)	39.87 (0.07)	32.01 (7.85)	40.95 (6.12)	39.37 (8.55)	9.463‡	0.844	0.402
TTA (cm^2)	145.70 (18.92)	138.35 (20.87)	117.39 (19.70)	162.20 (47.44)	156.65 (37.68)	149.35 (52.91)	3.983	1.033	0.166	139.90 (17.06)	118.80 (11.04)	127.91 (55.29)	128.77 (32.92)	145.35 (46.41)	156.29 (37.47)	167.08 (41.73)	153.13 (42.46)	4.258*	0.103	0.461
TMA (cm^2)	132.42 (18.64)	125.29 (20.76)	104.66 (19.96)	149.98 (38.22)	143.53 (38.35)	137.70 (54.28)	4.103	0.938	0.173	126.78 (17.10)	105.71 (10.27)	114.98 (53.99)	116.27 (33.81)	132.28 (45.46)	143.17 (39.00)	155.29 (42.70)	140.69 (43.21)	4.256*	0.114	0.464
TFA (cm^2)	13.29 (1.13)	13.06 (1.19)	12.72 (1.26)	12.23 (1.22)	13.13 (1.39)	11.65 (1.47)	2.285	1.291	0.802	13.12 (1.32)	13.08 (0.91)	12.93 (1.30)	12.50 (0.89)	13.08 (1.00)	13.12 (1.69)	11.79 (1.51)	12.44 (0.85)	0.330	0.594	0.274
TFI (%)	9.23 (1.28)	9.62 (1.75)	11.12 (2.20)	8.00 (2.57)	8.85 (2.35)	8.66 (3.65)	3.451	0.753	0.370	9.52 (1.64)	11.04 (0.56)	10.91 (3.70)	10.12 (3.28)	9.49 (2.40)	8.85 (2.43)	7.60 (2.81)	8.69 (3.14)	3.640	0.112	0.664
%F	15.17 (4.02)	15.12 (3.65)	12.72 (4.30)	19.78 (6.14)	16.14 (6.03)	17.56 (3.55)	3.953	0.648	0.568	15.17 (3.51)	12.34 (0.58)	12.08 (5.00)	15.01 (9.15)	16.98 (2.87)	15.71 (5.93)	19.11 (4.95)	18.57 (9.57)	3.863	0.354	0.316
FM (kg)	7.09 (2.26)	6.46 (1.78)	4.65 (2.32)	8.47 (3.54)	6.49 (3.41)	7.34 (4.04)	1.747	1.032	0.554	6.44 (1.74)	4.99 (0.85)	4.67 (3.34)	7.04 (5.84)	6.50 (2.71)	6.35 (3.38)	8.55 (3.59)	7.43 (5.00)	1.420	0.276	0.513
FFM (kg)	39.41 (3.85)	35.87 (4.59)	30.35 (3.86)	33.23 (4.14)	31.63 (4.52)	32.66 (11.12)	2.231	2.144	1.739	35.65 (4.65)	35.68 (7.67)	31.33 (9.39)	35.96 (6.89)	30.83 (8.04)	32.65 (3.24)	34.75 (6.81)	29.24 (4.74)	1.492	0.083	0.828

R (Ω)	607.75 (77.22)	631.10 (60.77)	681.83 (84.04)	731.39 (86.33)	717.00 (121.95)	750.67 (68.45)	8.256‡	0.735	0.240	624.41 (58.92)	695.33 (123.06)	646.05 (153.37)	638.75 (18.74)	673.82 (183.09)	771.16 (82.26)	742.90 (67.66)	684.38 (65.19)	3.566	1.351	0.111
Xc (Ω)	59.82 (7.38)	60.00 (5.16)	70.50 (8.02)	73.65 (5.20)	72.28 (8.70)	74.82 (4.13)	16.003†	2.384	1.168	63.29 (7.82)	69.07 (14.02)	59.30 (4.10)	59.95 (0.49)	68.68 (13.55)	76.05 (4.96)	74.45 (4.02)	70.82 (3.93)	9.717‡	1.279	0.527
PA	5.62 (0.17)	5.45 (0.47)	5.933 (0.89)	5.86 (0.33)	5.86 (0.37)	5.78 (0.27)	0.903	0.430	0.829	5.79 (0.67)	5.63 (0.15)	5.35 (0.92)	5.35 (0.07)	5.96 (0.51)	5.70 (0.32)	5.80 (0.26)	6.04 (0.25)	2.990	0.479	0.460
R/H ((Ω/cm)	382.53 (51.13)	410.26 (48.15)	470.50 (65.03)	506.70 (56.77)	506.60 (89.53)	532.22 (57.45)	15.859†	1.809	0.519	407.88 (47.44)	450.85 (101.70)	453.56 (134.58)	416.71 (45.28)	481.13 (140.74)	539.67 (54.28)	511.66 (51.33)	494.31 (61.79)	6.962*	0.831	0.051
Xc/H ((Ω/cm)	37.67 (5.07)	38.99 (4.10)	48.55 (5.24)	51.05 (3.40)	51.06 (6.64)	53.03 (4.03)	29.378†	4.258*	1.960	41.43 (6.56)	44.83 (11.35)	41.42 (5.40)	39.08 (3.43)	48.98 (10.87)	53.25 (3.36)	51.26 (3.24)	51.12 (4.40)	13.712†	0.585	0.140
CMJ (cm)	27.75 (2.95)	28.68 (0.97)	27.55 (4.20)	19.96 (4.26)	27.71 (3.58)	21.03 (2.65)	18.026†	6.187‡	3.590*	27.32 (2.81)	29.60 (2.69)	30.25 (1.20)	27.05 (4.60)	22.53 (4.71)	27.04 (3.41)	23.38 (6.82)	21.63 (4.82)	9.152‡	1.334	0.314
Sprint 15 meters (s)	2.73 (0.14)	2.68 (0.10)	2.73 (0.12)	3.24 (0.24)	3.08 (0.11)	3.35 (0.17)	97.685†	3.794*	1.761	2.72 (0.12)	2.67 (0.04)	2.63 (0.04)	2.78 (0.23)	3.35 (0.17)	3.03 (0.02)	3.18 (0.21)	3.25 (0.18)	66.984†	2.992	1.317
RSA (s)	6.36 (0.27)	6.27 (0.13)	6.39 (0.24)	7.30 (0.66)	7.01 (0.28)	7.55 (0.24)	65.095†	2.866	1.141	6.37 (0.23)	6.21 (0.16)	6.26 (0.03)	6.51 (0.35)	7.69 (0.07)	6.98 (0.31)	7.23 (0.50)	7.18 (0.51)	44.826†	2.443	1.213

Note: E, early; OT, on time; L, late; Q1, quartile one; Q2, quartile two; Q3, quartile three; Q4, quartile four; Bo, Bologna F.C.; Ru, Russi: S. U.; MS, maturity status; RAE, relative age effect; SD, standard deviation; F, Snedecor-Fischer statistic test; BMI, body mass index; circ., circumference; SK, skinfold thickness; TUA, total upper area; UMA, upper muscle area; UFA, upper-fat area; UFI, upper-fat index; TCA, total calf area; CMA, calf mass area; CFA, calf fat area; CFI, calf fat index; TTA, total thigh area; TMA, thigh mass area; TFA, thigh fat area; TFI, thigh fat index; %F, fat percentage; FM, fat mass; FFM, fat-free mass; R, resistance; Xc, reactance; PA, phase angle; CMJ, counter-movement jump; RSA, repeated sprint abiliy; *, p-value ≤ 0.05; ‡, p-value ≤ 0.01; †, p-value ≤ 0.001.
