# Supplementary material for: Maturation Selection Biases and Relative Age Effect in Italian Soccer Players of Different Levels
Source: Biology (Basel). 2022 Oct 24;11(11):1559. doi: 10.3390/biology11111559 (PMC9687510; doi:10.3390/biology11111559)
Supplement: Supplementary file 1 [file biology-11-01559-s001.zip › Table S2.pdf]

Table S2. Variables mean comparisons and interaction effects of Maturity Status, Teams and RAE in U13 soccer players.

	U13 Bologna			U13 Russi						U13 Bologna				U13 Russi						
	E (n=7)	OT (n=9)	L (n=11)	E (n=2)	OT (n=8)	L (n=2)	Bo-Ru	MS	teams*MS	Q1 (n=12)	Q2 (n=5)	Q3 (n=8)	Q4 (n=2)	Q1 (n=3)	Q2 (n=3)	Q3 (n=2)	Q4 (n=4)	Bo-Ru	RAE	teams*RAE
	Mean (± SD)	Mean (± SD)	Mean (± SD)	Mean (± SD)	Mean (± SD)	Mean (± SD)	$F_{(1, 37)}$	$F_{(2, 36)}$	$F_{(5, 33)}$	Mean (± SD)	Mean (± SD)	Mean (± SD)	Mean (± SD)	Mean (± SD)	Mean (± SD)	Mean (± SD)	Mean (± SD)	$F_{(1, 36)}$	$F_{(3, 34)}$	$F_{(7, 30)}$
Weight (Kg)	50.14 (6.77)	43.44 (4.25)	40.27 (3.82)	47.50 (7.07)	51.88 (5.36)	35.50 (9.19)	0.026	9.869†	5.063*	42.58 (4.72)	48.00 (8.80)	43.63 (6.63)	42.50 (3.54)	49.00 (3.50)	57.17 (4.07)	48.50 (3.54)	41.38 (9.45)	4.108*	3.321*	0.735
Height (cm)	162.63 (7.41)	156.7 (4.51)	148.66 (3.81)	162.80 (7.64)	155.44 (5.35)	138.85 (9.83)	2.690	20.650†	1.778	154.72 (6.51)	158.12 (10.69)	153.69 (8.00)	153.65 (8.56)	162.90 (6.68)	157.17 (3.04)	151.00 (2.26)	146.15 (10.66)	0.061	2.016	1.346
Trunk Height (cm)	81.66 (2.82)	78.11 (1.55)	74.36 (2.05)	83.00 (0.57)	78.91 (1.48)	69.95 (3.32)	0.853	42.778†	4.619*	77.67 (3.00)	77.72 (4.32)	77.20 (4.43)	77.20 (5.37)	81.90 (1.41)	78.33 (0.57)	78.30 (0.28)	74.98 (6.47)	0.393	1.085	0.837
Leg length (cm)	80.97 (6.6)	78.59 (3.99)	74.30 (3.53)	79.80 (7.07)	76.53 (4.44)	68.90 (6.51)	2.253	6.057‡	0.392	77.05 (4.54)	80.40 (8.53)	76.49 (4.44)	76.45 (3.18)	81.00 (5.41)	78.83 (3.07)	72.70 (1.98)	71.18 (4.71)	0.731	2.291	1.221
BMI (kg/m²)	18.93 (1.99)	17.66 (1.04)	18.21 (1.48)	17.86 (0.99)	21.52 (2.50)	18.21 (2.18)	1.683	1.974	5.744‡	17.78 (1.52)	19.09 (2.21)	18.38 (1.14)	18.00 (0.51)	18.46 (0.44)	23.17 (1.98)	21.30 (2.19)	19.21 (3.02)	11.130‡	4.661‡	1.559
Relaxed arm circ. (cm)	22.27 (1.6)	21.30 (1.30)	20.45 (1.47)	21.70 (1.13)	24.88 (1.81)	21.35 (1.63)	4.438*	5.192‡	4.855‡	20.68 (1.47)	22.04 (2.13)	21.68 (1.32)	20.40 (0.85)	22.33 (0.47)	26.13 (0.90)	24.95 (0.78)	22.45 (2.68)	20.687†	5.054‡	0.958
Contracted arm circ. (cm)	24.57 (1.74)	23.37 (1.37)	22.30 (1.59)	23.85 (1.63)	26.26 (1.69)	23.40 (2.12)	2.811	3.598*	3.040	22.81 (1.81)	23.96 (2.13)	23.68 (1.62)	22.35 (0.64)	24.57 (0.67)	27.53 (1.34)	25.60 (0.99)	24.28 (2.55)	11.486‡	2.651	0.439
Calf circ. (cm)	33.56 (3.24)	30.43 (5.73)	29.69 (1.90)	32.65 (1.91)	34.36 (1.26)	29.40 (1.56)	0.442	2.210	1.646	30.77 (4.18)	31.90 (2.98)	29.49 (3.92)	35.40 (5.66)	33.67 (0.31)	35.17 (1.69)	34.00 (1.27)	31.13 (2.53)	1.387	0.391	1.771
Thigh circ. (cm)	42.40 (6.31)	40.11 (3.25)	40.45 (1.96)	43.65 (1.77)	48.54 (2.93)	42.00 (4.81)	6.317*	1.742	3.345*	40.30 (3.19)	44.00 (3.18)	41.20 (2.31)	34.80 (8.20)	45.07 (1.26)	50.43 (1.76)	47.25 (0.64)	44.65 (5.75)	26.744†	5.324‡	0.634
Humeral diameter (mm)	6.33 (0.43)	6.16 (0.28)	5.94 (0.22)	6.25 (0.07)	6.21 (0.42)	5.35 (0.49)	2.255	7.832‡	2.256	6.08 (0.38)	6.04 (0.36)	6.19 (0.25)	6.15 (0.49)	6.13 (0.50)	6.33 (0.25)	5.90 (0.28)	5.93 (0.74)	0.074	0.195	0.730
Femoral diameter (mm)	8.97 (0.55)	8.56 (0.49)	8.42 (0.41)	7.65 (1.77)	9.45 (0.49)	8.35 (0.78)	0.511	4.837*	8.819†	8.55 (0.47)	8.74 (0.59)	8.64 (0.60)	8.50 (0.42)	9.40 (0.78)	9.60 (0.10)	9.15 (0.64)	8.08 (1.27)	3.300	2.051	1.318
Triceps SK (mm)	7.43 (1.43)	8.06 (1.94)	8.91 (1.30)	6.50 (0.71)	12.38 (3.54)	11.00 (4.24)	4.071	4.984*	3.207	8.38 (1.61)	8.10 (2.30)	8.31 (1.58)	7.50 (0.71)	7.67 (1.53)	12.33 (3.06)	15.00 (4.24)	11.00 (4.08)	15.762†	3.540*	3.764*
Biceps SK (mm)	4.14 (0.94)	4.83 (1.37)	4.86 (1.63)	4.25 (0.35)	8.44 (3.35)	7.00 (2.83)	5.740*	3.341*	1.762	4.63 (1.48)	4.40 (1.39)	5.13 (1.36)	3.75 (1.06)	4.00 (1.00)	9.17 (2.47)	11.00 (2.83)	7.13 (2.78)	26.243†	6.901†	5.760‡
Subscapular SK (mm)	6.29 (0.99)	4.94 (0.39)	5.23 (0.85)	6.25 (1.06)	9.81 (3.52)	7.50 (2.12)	10.599‡	1.281	4.552*	5.04 (0.54)	5.90 (1.14)	5.69 (1.19)	5.25 (0.35)	6.33 (0.58)	11.67 (4.73)	10.25 (1.06)	7.88 (2.59)	31.771†	5.662‡	2.927*

Supraspinal SK (mm)	5.29 (0.91)	4.67 (1.25)	4.64 (1.19)	5.00 (1.41)	9.63 (4.27)	7.75 (2.47)	8.127‡	1.887	3.107	4.63 (1.00)	5.20 (1.25)	5.00 (1.41)	4.25 (0.35)	5.00 (1.00)	11.33 (4.04)	9.50 (2.12)	8.63 (4.82)	22.483†	3.997*	2.876
Suprailiac SK (mm)	7.93 (1.79)	6.72 (2.08)	7.45 (1.75)	8.50 (0.71)	12.63 (4.37)	11.75 (4.6)	10.850‡	0.703	2.226	6.92 (1.84)	8.20 (2.28)	7.50 (1.85)	7.00 (1.41)	7.67 (1.15)	14.67 (3.51)	12.50 (2.12)	12.38 (4.92)	21.862†	4.184‡	2.238
Thigh SK (mm)	8.43 (1.27)	9.33 (1.41)	11.05 (1.52)	9 .00 (1.00)	13.50 (4.00)	11.00 (4.24)	2.763	3.118	2.629	9.83 (1.80)	9.50 (1.87)	10.13 (1.73)	9.00 (2.83)	8.33 (1.15)	14.67 (2.52)	15.50 (3.54)	12.00 (4.24)	12.055‡	4.276‡	4.203‡
Medial Calf SK (mm)	6.79 (1.29)	6.33 (1.64)	7.73 (2.41)	6.50 (0.71)	10.81 (2.42)	8.00 (1.41)	3.365	2.126	4.593*	7.08 (2.04)	7.20 (1.64)	7.06 (2.41)	6.00 (1.10)	7.50 (0.87)	11.00 (1.73)	12.50 (2.12)	8.75 (3.10)	14.846†	2.478	1.991
Lateral Calf SK (mm)	7.14 (1.65)	7.11 (1.02)	8.27 (2.23)	7.50 (0.71)	11.00 (2.62)	8.75 (2.47)	3.881	1.754	2.830	7.88 (1.92)	7.20 (1.92)	7.75 (1.77)	6.25 (0.35)	7.67 (0.58)	12.00 (1.73)	11.50 (2.12)	9.63 (3.35)	14.961†	1.937	2.611
TUA (cm^2)	39.67 (5.7)	36.24 (4.55)	33.44 (4.75)	37.54 (3.91)	49.49 (7.01)	36.40 (5.53)	4.566*	5.501‡	5.388‡	34.19 (4.78)	38.97 (7.42)	37.53 (4.72)	33.16 (2.76)	39.72 (1.67)	54.42 (3.69)	49.59 (3.09)	40.56 (9.95)	22.372†	5.652‡	1.266
UMA (cm^2)	31.63 (5.22)	28.78 (4.43)	26.54 (4.45)	29.43 (3.24)	42.31 (7.23)	29.85 (6.31)	5.145*	5.348‡	5.795‡	27.05 (4.42)	31.25 (6.97)	29.98 (4.18)	25.85 (2.23)	31.73 (1.27)	46.77 (3.49)	43.32 (4.46)	33.72 (10.29)	25.371†	6.044‡	1.708
UFA (cm^2)	8.03 (0.66)	7.46 (0.52)	6.90 (0.43)	8.12 (0.66)	7.19 (1.10)	6.54 (0.78)	0.426	6.114‡	0.194	7.14 (0.60)	7.72 (0.84)	7.55 (0.73)	7.31 (0.53)	8.00 (0.61)	7.64 (1.26)	6.27 (1.37)	6.84 (0.70)	0.704	1.688	2.629
UFI (%)	20.46 (2.04)	20.81 (2.39)	20.92 (2.33)	21.64 (0.48)	14.86 (3.45)	18.35 (4.92)	5.185*	3.665*	4.301*	21.14 (2.34)	20.20 (3.05)	20.21 (1.68)	22.06 (0.25)	20.12 (1.03)	14.05 (2.11)	12.75 (3.56)	17.70 (4.98)	22.356†	4.474‡	2.306
TCA (cm^2)	90.37 (18.00)	76.07 (29.79)	70.45 (8.85)	85.02 (9.93)	94.12 (6.96)	68.91 (7.28)	0.272	2.224	1.368	76.64 (23.29)	81.59 (15.27)	70.30 (16.98)	101.05 (31.89)	90.25 (1.64)	98.61 (9.39)	92.10 (6.89)	77.51 (12.65)	1.046	0.380	1.790
CMA (cm^2)	68.51 (15.75)	56.94 (25.19)	48.64 (6.66)	63.64 (6.59)	60.38 (5.51)	46.4 (1.29)	0.042	2.619	0.214	55.3 (19.79)	60.02 (9.85)	50.30 (15.54)	80.49 (27.86)	66.53 (2.04)	62.25 (5.14)	55.76 (0.25)	51.32 (6.15)	0.191	0.724	2.104
CFA (cm^2)	21.86 (5.09)	19.12 (5.88)	21.81 (6.60)	21.38 (3.33)	33.74 (7.79)	22.51 (6.00)	3.689	1.872	4.761*	21.34 (6.39)	21.57 (6.71)	20.00 (6.04)	20.55 (4.02)	23.72 (1.87)	36.37 (6.45)	36.34 (7.14)	26.19 (10.42)	14.958†	1.848	2.032
CFI (%)	24.41 (4.57)	26.34 (5.96)	30.65 (7.10)	25.08 (0.99)	35.64 (6.69)	32.39 (5.28)	2.450	2.666	1.582	28.18 (6.31)	26.16 (4.31)	29.33 (8.02)	20.75 (2.57)	26.28 (1.98)	36.75 (3.79)	39.28 (4.81)	33.09 (8.31)	10.357‡	2.154	2.219
TTA (cm^2)	145.85 (38.14)	128.84 (19.66)	130.58 (13.17)	151.82 (12.29)	188.17 (22.61)	141.37 (32.16)	7.096*	2.259	4.297*	130.05 (19.92)	154.78 (22.18)	135.52 (15.61)	99.10 (45.45)	161.79 (9.05)	202.67 (14.01)	177.77 (4.79)	160.7 (41.85)	28.164†	5.417‡	0.523
TMA (cm^2)	130.81 (36.13)	115.10 (18.06)	117.80 (12.54)	136.65 (11.66)	174.73 (24.09)	128.22 (33.30)	7.466‡	2.324	4.642*	116.56 (18.64)	139.79 (20.66)	121.88 (13.98)	87.27 (43.99)	145.67 (9.23)	189.51 (15.29)	166.03 (7.20)	147.5 (42.97)	29.712†	5.586‡	0.647
TFA (cm^2)	15.04 (2.16)	13.74 (1.76)	12.78 (1.12)	15.17 (0.63)	13.44 (2.28)	13.14 (1.14)	0.008	2.562	0.098	13.49 (1.70)	14.99 (1.90)	13.64 (1.80)	11.83 (1.46)	16.12 (0.48)	13.17 (1.52)	11.74 (2.41)	13.20 (1.36)	0.013	3.161*	3.629*
TFI (%)	10.83 (2.43)	10.74 (0.74)	9.83 (0.87)	10.01 (0.39)	7.32 (2.00)	9.64 (3.00)	5.211*	1.832	3.126	10.46 (1.06)	9.72 (0.81)	10.06 (0.54)	12.96 (4.47)	9.99 (0.73)	6.55 (1.19)	6.62 (1.53)	8.75 (2.82)	25.376†	5.696‡	2.462

%F	13.36 (1.97)	12.64 (2.24)	13.78 (1.78)	12.43 (0.36)	20.92 (5.43)	17.79 (5.82)	8.616‡	3.384*	4.961‡	13.07 (1.90)	13.63 (2.41)	13.64 (2.23)	12.43 (0.36)	13.67 (0.99)	22.47 (5.58)	23.64 (4.27)	18.02 (6.01)	28.945†	5.213‡	3.953*
FM (kg)	6.75 (1.59)	5.52 (1.31)	5.58 (1.11)	5.92 (1.05)	10.97 (3.49)	6.58 (3.70)	5.004*	3.610*	6.409‡	5.60 (1.22)	6.59 (1.95)	5.95 (1.34)	5.27 (0.29)	6.67 (0.02)	12.88 (3.58)	11.54 (2.91)	7.75 (3.97)	25.255†	5.686‡	3.030*
FFM (kg)	43.39 (5.48)	37.92 (3.43)	34.69 (2.97)	41.58 (6.02)	40.91 (3.91)	28.92 (5.49)	0.884	12.793†	3.049	36.98 (3.81)	41.41 (7.26)	37.67 (5.77)	37.23 (3.25)	42.33 (3.5)	44.28 (4.05)	36.96 (0.63)	33.63 (6.35)	0.257	2.611	1.058
R (Ω)	563.35 (34.04)	577.82 (55.01)	615.18 (69.45)	557.90 (75.24)	565.53 (42.42)	689.80 (40.02)	0.706	6.062‡	1.553	592.98 (63.14)	612.65 (79.13)	576.49 (58.93)	584.6 (13.44)	532.27 (70.14)	570.83 (8.88)	580.35 (49.14)	637.40 (68.18)	0.234	0.777	1.125
Xc (Ω)	62.70 (7.64)	71.04 (11.28)	69.84 (7.18)	56.60 (7.35)	58.61 (5.81)	75.15 (4.17)	1.755	4.082*	2.871	73.78 (10.19)	63.85 (6.51)	64.59 (5.76)	63.15 (1.34)	52.80 (3.99)	62.37 (2.35)	58.50 (10.04)	67.48 (9.33)	3.886	0.217	3.593*
PA	6.42 (0.80)	7.06 (1.20)	6.54 (0.84)	5.80 (0.65)	5.90 (0.40)	6.20 (0.50)	4.167*	0.424	0.646	7.19 (1.16)	6.00 (0.9)	6.40 (0.23)	6.2 (0.28)	5.63 (0.29)	6.23 (0.23)	5.75 (0.49)	6.03 (0.21)	3.202	0.340	1.984
R/H ((Ω/cm)	346.12 (29.89)	369.04 (37.22)	414.14 (49.43)	344.15 (62.36)	364.72 (35.75)	497.02 (6.36)	2.378	14.728†	2.967	383.93 (44.64)	392.72 (83.47)	376.74 (49.09)	380.82 (12.46)	328.23 (57.00)	363.35 (11.99)	384.62 (38.31)	439.03 (67.32)	0.056	1.234	1.505
Xc/H ((Ω/cm)	38.46 (4.48)	45.29 (6.71)	46.96 (4.45)	34.91 (6.15)	37.78 (4.29)	54.15 (0.83)	0.386	12.735†	5.064‡	47.66 (6.09)	40.69 (5.62)	42.22 (5.30)	41.19 (3.17)	32.51 (3.78)	39.68 (1.25)	38.80 (7.23)	46.56 (8.79)	2.495	0.566	4.119*
CMJ (cm)	28.66 (6.77)	29.84 (2.84)	27.64 (2.72)	28.20 (7.92)	23.46 (4.55)	23.40 (2.55)	4.408*	0.735	1.038	27.73 (4.60)	30.12 (3.38)	28.58 (1.49)	29.95 (6.15)	30.13 (3.56)	24.90 (2.45)	19.55 (2.47)	21.68 (3.29)	11.936‡	2.219	3.787*
Sprint 15 meters (s)	2.82 (0.18)	2.83 (0.07)	2.84 (0.08)	2.80 (0.21)	3.12 (0.14)	3.11 (0.16)	12.082‡	4.194*	3.507*	2.85 (0.08)	2.85 (0.18)	2.82 (0.10)	2.76 (0.01)	2.89 (0.24)	3.09 (0.07)	3.19 (0.09)	3.13 (0.19)	24.896†	1.494	2.659
RSA (s)	6.65 (0.34)	6.56 (0.13)	6.55 (0.12)	6.40 (0.30)	7.04 (0.34)	6.98 (0.23)	5.199*	3.078	5.548‡	6.61 (0.21)	6.58 (0.27)	6.50 (0.10)	6.59 (0.13)	6.54 (0.38)	7.14 (016)	7.41 (0.12)	6.94 (0.28)	16.242†	2.842	4.301‡

Note: E, early; OT, on time; L, late; Q1, quartile one; Q2, quartile two; Q3, quartile three; Q4, quartile four; Bo, Bologna F.C.; Ru, Russi: S. U.; MS, maturity status; RAE, relative age effect; SD, standard deviation; F, Snedecor-Fischer statistic test; BMI, body mass index; circ., circumference; SK, skinfold thickness; TUA, total upper area; UMA, upper muscle area; UFA, upper-fat area; UFI, upper-fat index; TCA, total calf area; CMA, calf mass area; CFA, calf fat area; CFI, calf fat index; TTA, total thigh area; TMA, thigh mass area; TFA, thigh fat area; TFI, thigh fat index; %F, fat percentage; FM, fat mass; FFM, fat-free mass; R, resistance; Xc, reactance; PA, phase angle; CMJ, counter-movement jump; RSA, repeated sprint ability; *, p-value ≤ 0.05; ‡, p-value ≤ 0.01; †, p-value ≤ 0.001.
