# Supplementary material for: Maturation Selection Biases and Relative Age Effect in Italian Soccer Players of Different Levels
Source: Biology (Basel). 2022 Oct 24;11(11):1559. doi: 10.3390/biology11111559 (PMC9687510; doi:10.3390/biology11111559)
Supplement: Supplementary file 1 [file biology-11-01559-s001.zip › Table S3.pdf]

Table S3. Variables mean comparisons and interaction effects of Maturity Status, Teams and RAE in U14 soccer players.

	U14 Bologna			U14 Russi						U14 Bologna				U14 Russi						
	E (n=9)	OT (n=14)	L (n=7)	E (n=6)	OT (n=9)	L (n=6)	Bo-Ru	MS	teams*MS	Q1 (n=17)	Q2 (n=6)	Q3 (n=6)	Q4 (n=1)	Q1 (n=6)	Q2 (n=6)	Q3 (n=6)	Q4 (n=1)	Bo-Ru	RAE	teams*RAE
	Mean (± SD)	Mean (± SD)	Mean (± SD)	Mean (± SD)	Mean (± SD)	Mean (± SD)	F $_{(1, 47)}$	F $_{(2, 46)}$	F $_{(5, 43)}$	Mean (± SD)	Mean (± SD)	Mean (± SD)	Mean	Mean (± SD)	Mean (± SD)	Mean (± SD)	Mean	F $_{(1, 47)}$	F $_{(3, 45)}$	F $_{(7, 41)}$
Weight (Kg)	62.00 (4.86)	53.04 (6.57)	45.89 (4.31)	61.92 (10.21)	54.44 (5.82)	39.92 (5.40)	0.705	31.591†	1.549	54.38 (8.44)	49.67 (7.45)	53.17 (8.16)	48.00	57.75 (7.21)	13.63 (7.21)	47.08 (10.66)	38.50	0.33	2.089	0.988
Height (cm)	175.66 (7.68)	165.99 (4.15)	158.22 (5.18)	170.37 (5.37)	160.37 (4.9)	152.33 (3.60)	13.821†	40.438†	0.012	166.92 (8.71)	165.72 (8.81)	164.25 (8.00)	160.00	166.19 (7.87)	4.83 (7.87)	159.55 (8.12)	146.60	3.887	2.456	0.78
Trunk Height (cm)	90.73 (2.68)	84.06 (2.07)	79.44 (2.88)	89.05 (2.24)	81.89 (2.44)	77.12 (2.06)	8.607‡	80.241†	0.069	85.36 (5.14)	83.90 (4.64)	81.62 (3.77)	82.80	85.36 (5.03)	3.37 (5.03)	80.98 (5.72)	75.60	1.549	2.538	0.433
Leg length (cm)	84.93 (7.65)	81.92 (3.30)	78.78 (3.67)	81.32 (3.47)	78.48 (2.84)	75.22 (3.41)	8.411‡	7.373‡	0.002	81.56 (5.59)	81.82 (4.99)	82.63 (4.51)	77.20	80.83 (3.29)	2.85 (3.29)	78.57 (2.97)	71.00	4.769*	1.885	0.952
BMI (kg/m^2)	20.09 (0.84)	19.19 (1.62)	18.32 (1.41)	21.38 (3.93)	21.20 (2.44)	17.20 (2.17)	1.37	8.129†	2.448	19.38 (1.34)	17.99 (0.77)	19.66 (2.21)	18.75	20.93 (2.63)	4.20 (2.63)	18.34 (3.10)	17.91	0.408	0.85	1.969
Relaxed arm circ. (cm)	24.29 (1.00)	23.06 (2.10)	21.71 (1.57)	25.28 (2.72)	24.97 (2.46)	21.57 (2.30)	2.288	8.875†	1.06	23.39 (1.99)	21.95 (1.85)	22.92 (1.85)	21.30	24.64 (2.39)	3.28 (2.39)	23.13 (3.33)	21.60	1.253	1.001	0.609
Contracted arm circ. (cm)	26.76 (1.45)	25.28 (1.96)	23.61 (1.90)	27.38 (2.66)	27.42 (3.17)	22.82 (2.37)	0.969	11.68†	1.82	25.58 (2.28)	24.08 (1.27)	25.12 (2.34)	23.60	27.26 (3.43)	3.41 (3.43)	24.75 (3.48)	22.80	0.387	1.477	0.653
Calf circ. (cm)	37.36 (8.09)	33.33 (1.87)	32.39 (2.09)	35.48 (2.39)	34.69 (1.7)	30.40 (2.33)	0.663	7.041‡	1.346	33.49 (1.65)	35.78 (9.81)	34.02 (2.3)	31.40	35.23 (1.80)	3.11 (1.80)	31.98 (3.38)	30.90	0.17	0.817	0.839
Thigh circ. (cm)	47.53 (2.38)	45.31 (3.89)	43.07 (2.96)	50.88 (4.84)	49.20 (4.39)	41.93 (4.83)	3.166	11.007†	1.954	45.69 (3.56)	42.65 (3.15)	46.45 (3.79)	43.30	49.76 (5.08)	6.09 (5.08)	44.80 (6.45)	44.70	1.417	1.128	1.419
Humeral diameter (mm)	6.64 (0.38)	6.49 (0.22)	6.11 (0.22)	6.80 (0.38)	6.44 (0.27)	6.07 (0.33)	0.064	16.731†	0.592	6.42 (0.37)	6.52 (0.33)	6.32 (0.21)	6.20	6.65 (0.33)	0.47 (0.33)	6.28 (0.44)	6.10	0.008	1.487	0.788
Femoral diameter (mm)	9.49 (0.45)	9.41 (0.40)	8.89 (0.65)	9.78 (0.39)	9.41 (0.35)	8.83 (0.56)	0.355	10.118†	0.559	9.26 (0.62)	9.27 (0.41)	9.38 (0.50)	8.80	9.49 (0.30)	0.70 (0.30)	9.33 (0.68)	8.60	0.001	0.904	0.221
Triceps SK (mm)	5.86 (1.60)	7.21 (1.40)	7.44 (2.52)	9.17 (2.48)	10.00 (4.35)	10.00 (3.16)	13.511†	0.87	0.071	6.62 (1.75)	7.50 (1.38)	7.42 (2.80)	7.00	9.56 (3.16)	3.53 (3.16)	10.83 (3.87)	13.00	8.728‡	0.746	0.792

Biceps SK (mm)	4.36 (1.25)	4.43 (1.24)	4.89 (1.60)	5.08 (2.06)	7.17 (3.73)	5.67 (2.16)	5.113*	1.048	1.31	4.71 (1.43)	4.17 (0.98)	4.58 (1.56)	4.00	5.69 (3.47)	2.82 (3.47)	6.50 (3.03)	7.00	4.370*	0.073	0.264
Subscapular SK (mm)	6.57 (0.73)	6.21 (1.09)	5.61 (0.89)	8.50 (5.17)	9.44 (4.16)	6.17 (1.03)	6.320*	2.598	1.168	6.18 (0.92)	5.92 (0.66)	6.33 (1.51)	5.00	7.88 (3.36)	5.75 (3.36)	7.58 (3.26)	6.50	3.125	0.426	0.503
Supraspinal SK (mm)	4.86 (0.85)	5.00 (1.00)	4.83 (1.48)	8.67 (5.71)	8.44 (3.50)	6.00 (2.81)	12.033†	1.163	0.99	4.88 (1.05)	4.75 (0.88)	5.10 (1.71)	5.50	7.56 (3.37)	6.12 (3.37)	7.17 (3.27)	8.50	5.601*	0.129	0.208
Suprailiac SK (mm)	8.07 (1.54)	7.96 (1.99)	7.78 (2.43)	11.08 (4.54)	11.94 (5.34)	9.17 (3.49)	8.134‡	0.87	0.651	7.97 (2.00)	7.00 (1.45)	8.92 (2.31)	7.00	10.63 (4.24)	6.71 (4.24)	11.58 (3.53)	9.00	3.705	0.422	0.111
Thigh SK (mm)	8.29 (1.11)	9.29 (1.89)	9.94 (2.60)	11.42 (4.74)	13.33 (4.90)	10.75 (4.59)	7.199‡	0.83	1.007	9.29 (1.58)	9.17 (1.33)	9.58 (3.58)	7.00	11.38 (4.03)	5.65 (4.03)	12.67 (5.60)	13.00	5.572*	0.148	0.217
Medial Calf SK (mm)	6.36 (1.11)	6.75 (2.01)	7.33 (2.59)	7.92 (3.72)	9.50 (3.39)	6.83 (2.32)	2.816	0.94	1.715	7.00 (1.88)	6.75 (1.08)	6.75 (3.19)	5.00	7.63 (2.99)	4.29 (2.99)	8.33 (3.27)	9.00	3.363	0.133	0.398
Lateral Calf SK (mm)	7.14 (0.90)	7.79 (2.16)	7.94 (3.15)	9.00 (3.85)	10.39 (3.43)	7.92 (2.29)	3.387	0.968	1.012	7.85 (1.92)	7.33 (1.40)	7.83 (3.87)	6.00	9.06 (2.98)	4.39 (2.98)	9.33 (3.39)	9.00	2.66	0.081	0.124
TUA (cm^2)	47.03 (3.91)	42.65 (7.78)	37.70 (5.62)	51.39 (11.68)	50.06 (9.91)	37.38 (8.26)	2.604	8.098†	0.992	43.87 (7.43)	38.59 (6.52)	42.04 (6.66)	36.12	48.73 (9.63)	13.58 (9.63)	43.34 (12.32)	37.15	1.333	0.929	0.581
UMA (cm^2)	37.68 (3.8)	34.24 (7.02)	29.92 (5.62)	42.91 (11.70)	42.07 (10.62)	30.47 (8.21)	3.723	6.343‡	0.894	35.13 (6.71)	30.69 (5.78)	33.80 (6.59)	28.32	40.63 (9.84)	13.68 (9.84)	36.25 (12.43)	31.09	1.888	0.674	0.396
UFA (cm^2)	9.35 (0.49)	8.41 (0.90)	7.78 (0.55)	8.47 (0.46)	7.99 (0.95)	6.92 (0.78)	10.565‡	14.635†	0.538	8.74 (0.95)	7.90 (0.85)	8.24 (0.65)	7.80	8.10 (0.77)	0.54 (0.77)	7.09 (0.89)	6.06	4.435*	3.514*	2.709
UFI (%)	19.97 (1.71)	20.02 (2.14)	20.99 (3.07)	17.05 (3.04)	16.74 (4.59)	19.11 (3.86)	8.623‡	1.374	0.221	20.19 (2.15)	20.69 (1.90)	20.02 (3.55)	21.60	17.18 (3.52)	4.44 (3.52)	17.49 (4.93)	16.31	5.816*	0.14	0.115
TCA (cm^2)	115.57 (57.74)	88.70 (9.99)	83.83 (10.87)	100.62 (13.71)	96.01 (9.42)	73.94 (11.27)	0.732	5.332‡	1.111	89.52 (8.69)	108.34 (67.89)	92.48 (12.24)	78.50	99.02 (9.98)	17.28 (9.98)	82.2 (16.83)	76.02	0.209	0.752	0.784
CMA (cm^2)	91.57 (50.72)	66.02 (6.57)	60.94 (9.42)	72.70 (8.73)	64.54 (6.08)	53.05 (6.64)	2.65	5.704‡	0.785	66.41 (7.86)	84.53 (59.96)	69.24 (9.00)	62.18	71.79 (8.23)	4.99 (8.23)	55.95 (9.05)	50.75	1.271	0.705	1.159
CFA (cm^2)	24.00 (7.47)	22.67 (6.74)	22.89 (8.99)	27.92 (13.28)	31.47 (11.04)	20.90 (7.11)	1.825	1.477	1.551	23.11 (5.84)	23.80 (8.46)	23.24 (11.2)	16.32	27.22 (9.82)	15.24 (9.82)	26.25 (11.34)	25.27	1.743	0.224	0.072
CFI (%)	21.83 (3.48)	25.26 (5.73)	27.12 (8.29)	27.10 (9.47)	32.22 (8.99)	27.82 (6.18)	4.196*	1.442	0.834	25.8 (5.78)	24.00 (5.18)	24.51 (9.56)	20.79	27.19 (7.89)	10.82 (7.89)	31.02 (7.83)	33.24	4.415*	0.077	0.629
TTA (cm^2)	180.24 (18.53)	164.61 (28.51)	148.29 (20.89)	207.70 (41.06)	194.09 (36.52)	141.55 (32.54)	3.635	9.951†	1.784	167.19 (26.25)	145.48 (21.92)	172.74 (27.78)	149.27	198.96 (41.33)	49.94 (41.33)	162.56 (44.43)	159.08	1.514	1.075	1.314
TMA (cm^2)	163.15 (17.23)	149.09 (27.76)	134.02 (21.26)	192.06 (42.82)	180.44 (38.35)	128.35 (33.65)	4.145*	8.74†	1.785	151.49 (25.07)	130.80 (21.14)	157.07 (27.59)	132.93	183.60 (42.11)	52.08 (42.11)	149.87 (45.67)	146.26	1.814	0.928	1.153
TFA (cm^2)	17.09 (1.48)	15.52 (1.40)	14.27 (1.38)	15.63 (3.23)	13.65 (2.96)	13.20 (2.15)	5.653*	5.521‡	0.158	15.69 (1.67)	14.69 (1.12)	15.67 (2.39)	16.35	15.36 (2.78)	2.89 (2.78)	12.69 (2.97)	12.83	3.889	1.213	1.117
TFI (%)	9.50 (0.45)	9.61 (1.34)	9.79 (1.53)	7.83 (2.13)	7.35 (2.33)	9.84 (2.88)	5.816*	2.495	1.774	9.49 (0.94)	10.21 (1.05)	9.26 (1.95)	10.95	8.02 (1.94)	2.98 (1.94)	8.55 (3.44)	8.06	4.597*	0.166	0.33
%F	9.20 (1.13)	12.02 (2.34)	12.47 (3.31)	13.72 (6.01)	17.59 (7.27)	15.68 (3.44)	12.457†	2.538	0.329	10.90 (2.46)	11.62 (1.97)	13.06 (3.96)	11.67	14.65 (6.40)	7.07 (6.4)	16.75 (5.55)	18.85	6.394*	0.68	0.115

FM (kg)	5.73 (1.02)	6.44 (1.81)	5.76 (1.85)	8.86 (5.64)	9.87 (4.86)	6.34 (2.04)	6.708‡	1.944	0.971	5.86 (1.39)	5.75 (1.09)	7.04 (2.68)	5.60	8.50 (4.13)	6.27 (4.13)	8.14 (4.28)	7.26	2.522	0.101	0.29
FFM (kg)	56.27 (4.07)	46.60 (5.34)	40.13 (3.65)	53.06 (6.33)	44.58 (3.12)	33.58 (3.93)	8.718‡	52.157†	1.1	48.52 (8.02)	43.92 (6.87)	46.13 (6.61)	42.40	49.25 (6.98)	7.95 (6.98)	38.94 (7.87)	31.24	1.962	3.158*	1.009
R (Ω)	501.13 (36.72)	546.78 (58.73)	575.67 (60.91)	508 (40.74)	553.36 (33.67)	673.07 (73.38)	5.66*	18.003†	3.716*	527.78 (65.09)	575.15 (36.63)	548.98 (50.68)	626.70	541.16 (57.90)	58.85 (57.90)	631.43 (94.06)	691.80	1.447	3.729*	1.759
Xc (Ω)	57.61 (6.36)	58.47 (6.33)	60.07 (5.99)	57.42 (5.26)	59.02 (5.53)	67.28 (12.50)	1.512	3.127	1.259	58.22 (5.51)	58.67 (5.34)	58.67 (8.27)	68.80	58.85 (6.45)	7.08 (6.45)	64.82 (12.77)	64.50	0.059	1.127	0.539
PA	6.56 (0.53)	6.12 (0.46)	5.97 (0.35)	6.45 (0.34)	6.11 (0.66)	5.82 (1.17)	0.255	3.57*	0.064	6.32 (0.33)	5.82 (0.39)	6.10 (0.79)	6.30	6.24 (0.54)	0.64 (0.54)	5.98 (1.19)	5.30	0.53	0.776	0.973
R/H ((Ω/cm)	285.31 (17.56)	330.18 (41.51)	364.92 (48.32)	298.24 (23.31)	345.62 (27.78)	441.93 (48.05)	10.243‡	30.848†	3.538*	318.06 (52.28)	348.57 (36.31)	335.64 (42.61)	391.69	327.33 (48.53)	43.96 (48.53)	398.39 (74.95)	471.90	2.938	4.003‡	1.198
Xc/H ((Ω/cm)	32.81 (3.45)	35.29 (4.35)	38.08 (4.79)	33.69 (2.79)	36.88 (4.09)	44.18 (8.27)	4.221*	9.82†	1.313	35.03 (4.49)	35.57 (4.45)	35.77 (5.12)	43.00	35.52 (4.67)	5.05 (4.67)	40.88 (9.50)	44.00	0.88	1.881	0.468
Yo-Yo IRT (s)	2283.33 (548.22)	2445.00 (427.94)	2320.00 (693.04)	1135.00 (671.19)	602.22 (320.07)	844.00 (402.59)	82.904†	0.432	1.604	2316 (560.98)	2572 (345.14)	2240 (632.96)	2880.00	1062.86 (581.08)	284.44 (581.08)	572.00 (280.57)	0.00	0	1.109	1.662
CMJ (cm)	34.44 (2.53)	32.63 (2.64)	31.68 (3.52)	29.95 (4.75)	28.22 (6.83)	25.47 (6.89)	14.022†	2.12	0.19	33.16 (3.41)	31.47 (2.49)	33.43 (1.92)	29.90	30.23 (5.50)	5.54 (5.50)	24.52 (7.46)	23.20	7.539‡	1.477	1.428
Sprint 15 meters (s)	2.45 (0.09)	2.52 (0.09)	2.53 (0.08)	2.82 (0.22)	2.90 (0.22)	3.08 (0.22)	86.607†	4.029*	1.698	2.50 (0.08)	2.57 (0.03)	2.45 (0.14)	2.54	2.81 (0.18)	3.19 (0.18)	3.16 (0.17)	3.19	77.642†	4.147‡	7.101†
RSA (s)	5.77 (0.20)	5.87 (0.23)	5.84 (0.10)	6.47 (0.35)	6.61 (0.50)	6.82 (0.60)	52.180†	0.982	0.658	5.84 (0.11)	5.98 (0.14)	5.75 (0.32)	5.71	6.42 (0.41)	6.52 (0.35)	7.08 (0.52)	7.10	15.213†	3.179*	6.256‡

Note: E, early; OT, on time; L, late; Q1, quartile one; Q2, quartile two; Q3, quartile three; Q4, quartile four; Bo, Bologna F.C.; Ru, Russi: S. U.; MS, maturity status; RAE, relative age effect; SD, standard deviation; F, Snedecor-Fischer statistic test; BMI, body mass index; circ., circumference; SK, skinfold thickness; TUA, total upper area; UMA, upper muscle area; UFA, upper-fat area; UFI, upper-fat index; TCA, total calf area; CMA, calf mass area; CFA, calf fat area; CFI, calf fat index; TTA, total thigh area; TMA, thigh mass area; TFA, thigh fat area; TFI, thigh fat index; %F, fat percentage; FM, fat mass; FFM, fat-free mass; R, resistance; Xc, reactance; PA, phase angle; CMJ, counter-movement jump; RSA, repeated sprint ability; *, p-value ≤ 0.05; ‡, p-value ≤ 0.01; †, p-value ≤ 0.001.
