# Supplementary material for: Maturation Selection Biases and Relative Age Effect in Italian Soccer Players of Different Levels
Source: Biology (Basel). 2022 Oct 24;11(11):1559. doi: 10.3390/biology11111559 (PMC9687510; doi:10.3390/biology11111559)
Supplement: Supplementary file 1 [file biology-11-01559-s001.zip › Table S4.pdf]

Table S4. Variables mean comparisons and interaction effects of Maturity Status, Teams and RAE in U15 soccer players.

	U15 Bologna			U15 Russi						U15 Bologna				U15 Russi						
	E (n=9)	OT (n=8)	L (n=6)	E (n=5)	OT (n=5)	L (n=5)	Bo-Ru	MS	teams*MS	Q1 (n=11)	Q2 (n=5)	Q3 (n=3)	Q4 (n=4)	Q1 (n=6)	Q2 (n=3)	Q3 (n=1)	Q4 (n=5)	Bo-Ru	RAE	teams*RAE
	Mean (± SD)	Mean (± SD)	Mean (± SD)	Mean (± SD)	Mean (± SD)	Mean (± SD)	$F_{(1, 36)}$	$F_{(2, 35)}$	$F_{(5, 32)}$	Mean (± SD)	Mean (± SD)	Mean (± SD)	Mean (± SD)	Mean (± SD)	Mean (± SD)	Mean	Mean (± SD)	$F_{(1, 36)}$	$F_{(3, 35)}$	$F_{(7, 30)}$
Weight (Kg)	66.90 (9.18)	62.75 (4.56)	56.67 (10.95)	70.10 (15.70)	55.80 (8.87)	50.00 (4.00)	1.244	8.039†	1.193	63.55 (6.33)	65.00 (9.70)	62.33 (5.69)	58.25 (16.74)	58.08 (8.63)	74.00 (22.34)	56.00	50.60 (2.51)	0.414	3.042*	1.137
Height (cm)	179.92 (6.37)	171.69 (5.92)	165.50 (9.21)	178.04 (3.75)	167.68 (3.76)	163.60 (7.26)	1.469	15.622†	0.110	177.87 (7.23)	171.66 (5.32)	171.17 (1.02)	164.35 (13.66)	167.40 (9.21)	173.97 (10.10)	167.90	170.48 (6.26)	0.165	0.942	2.294
Trunk Height (cm)	92.89 (1.27)	87.46 (2.40)	82.40 (3.87)	91.36 (1.08)	86.66 (2.04)	83.02 (2.10)	0.560	50.554†	0.666	90.35 (2.83)	87.90 (4.23)	86.77 (4.82)	84.13 (8.37)	87.13 (4.77)	89.60 (3.84)	83.00	86.12 (2.75)	0.216	1.828	1
Leg length (cm)	87.03 (6.23)	84.23 (3.89)	83.10 (5.63)	86.68 (4.30)	81.02 (2.70)	80.58 (6.48)	1.394	3.383*	0.263	87.53 (5.52)	83.76 (3.04)	84.40 (4.47)	80.23 (5.38)	80.27 (5.29)	84.37 (6.44)	84.90	84.36 (5.04)	0.06	0.271	2.632
BMI (kg/m^2)	20.70 (2.94)	21.32 (1.75)	20.55 (3.15)	22.08 (4.67)	19.83 (2.92)	18.71 (1.44)	0.450	1.086	1.142	20.14 (2.15)	22.00 (2.53)	21.29 (2.15)	21.21 (4.01)	20.66 (2.03)	24.04 (5.02)	19.86	17.44 (1.05)	0.395	2.866	1.98
Relaxed arm circ. (cm)	24.87 (2.03)	24.05 (1.09)	23.85 (1.99)	26.92 (4.42)	24.28 (1.98)	22.42 (1.02)	0.146	4.68*	1.807	24.53 (1.75)	24.44 (1.56)	24.03 (1.33)	23.80 (2.59)	24.42 (2.11)	28.60 (5.02)	23.60	22.44 (1.02)	0.421	3.476*	2.478
Contracted arm circ. (cm)	26.92 (5.31)	26.64 (1.05)	26.30 (2.73)	28.66 (4.87)	25.96 (1.97)	24.62 (1.44)	0.031	1.377	0.768	26.54 (4.65)	26.80 (1.7)	27.33 (2.06)	26.33 (3.34)	26.58 (1.73)	30.73 (5.05)	24.90	23.92 (1.16)	0.025	1.619	1.355
Calf circ. (cm)	35.66 (2.27)	34.70 (1.70)	32.70 (2.51)	37.48 (3.54)	34.76 (2.89)	32.54 (1.20)	0.520	8.065†	0.639	34.89 (1.45)	35.44 (2.68)	33.43 (0.61)	33.35 (4.47)	35.65 (2.24)	37.80 (5.60)	33.60	32.60 (1.20)	0.367	3.08*	0.493
Thigh circ. (cm)	47.27 (3.91)	45.93 (4.67)	45.27 (2.84)	52.10 (6.71)	47.84 (5.21)	45.06 (3.62)	2.080	3.043	0.932	47.35 (2.02)	48.42 (4.45)	42.17 (6.12)	43.75 (2.87)	49.78 (3.49)	53.37 (9.15)	49.00	43.44 (2.72)	4.720*	5.244‡	0.893
Humeral diameter (mm)	6.90 (0.31)	6.63 (0.17)	6.55 (0.33)	6.92 (0.35)	6.64 (0.44)	6.42 (0.33)	0.090	5.609‡	0.207	6.76 (0.29)	6.82 (0.36)	6.70 (0.10)	6.45 (0.33)	6.55 (0.30)	6.80 (0.66)	6.60	6.72 (0.46)	0.012	0.559	0.868
Femoral diameter (mm)	9.72 (0.38)	9.43 (0.24)	8.97 (0.44)	10.18 (0.64)	9.56 (0.38)	9.46 (0.50)	6.54‡	9.254†	0.653	9.54 (0.29)	9.62 (0.53)	9.27 (0.31)	8.98 (0.64)	9.80 (0.28)	10.23 (1.10)	9.90	9.32 (0.28)	6.207*	4.154‡	0.316
Triceps SK (mm)	7.28 (2.24)	5.81 (1.60)	6.75 (1.25)	9.30 (4.35)	8.20 (3.27)	6.60 (2.16)	2.850	1.398	0.844	6.23 (1.71)	6.80 (3.05)	7.33 (1.15)	7.00 (0.82)	7.58 (2.11)	11.17 (4.75)	7.00	6.90 (3.54)	1.728	1.301	1.256
Biceps SK (mm)	3.78 (1.09)	3.50 (0.46)	3.67 (0.61)	5.80 (2.95)	5.10 (2.01)	4.40 (0.89)	9.024‡	0.843	0.598	3.55 (0.69)	3.70 (1.30)	3.67 (0.58)	3.88 (0.48)	4.50 (1.38)	7.67 (2.31)	5.00	4.30 (1.86)	11.099‡	3.293*	3.234*

Subscapular SK (mm)	7.17 (1.62)	6.38 (1.16)	6.50 (1.48)	8.30 (3.87)	7.30 (0.84)	5.20 (0.76)	0.173	3.153	1.567	6.77 (1.44)	6.50 (1.66)	7.00 (1.32)	6.63 (1.70)	6.25 (1.21)	10.17 (4.07)	6.00	6.00 (1.58)	0.285	2.247	2.865
Supraspinal SK (mm)	5.56 (1.33)	5.56 (1.12)	5.00 (0.84)	8.80 (4.38)	6.90 (3.05)	4.90 (0.65)	4.510*	3.302*	1.889	5.32 (1.12)	5.60 (1.39)	5.83 (1.04)	5.13 (1.18)	6.75 (2.72)	10.67 (4.93)	4.50	5.20 (1.15)	2.723	3.508*	2.915
Suprailiac SK (mm)	8.17 (2.03)	7.75 (2.14)	8.33 (2.07)	11.20 (4.49)	11.00 (2.55)	7.70 (1.96)	4.887*	1.388	2.084	7.73 (2.11)	8.20 (2.59)	9.00 (1.73)	8.13 (1.44)	9.50 (3.03)	13.33 (4.73)	9.50	8.60 (2.51)	3.628	1.472	1.275
Thigh SK (mm)	9.28 (2.48)	8.19 (1.56)	7.83 (1.29)	12.20 (6.30)	9.90 (3.03)	9.20 (2.59)	3.874	1.771	0.219	8.23 (1.42)	9.00 (3.66)	9.67 (0.58)	7.88 (0.63)	9.42 (2.11)	16.00 (5.20)	11.00	8.20 (3.40)	5.707*	4.831‡	2.804*
Medial Calf SK (mm)	7.06 (1.49)	5.50 (0.96)	5.00 (0.71)	9.40 (3.90)	7.20 (2.68)	6.20 (1.92)	6.699‡	5.423‡	0.239	5.95 (1.60)	6.00 (1.87)	6.00 (0.87)	6.00 (1.08)	7.50 (2.32)	11.00 (4.36)	7.00	5.80 (1.92)	5.049*	2.33	2.314
Lateral Calf SK (mm)	7.94 (1.57)	6.38 (0.99)	6.17 (0.98)	9.80 (4.42)	7.90 (2.70)	6.70 (2.08)	3.129	3.909*	0.280	7.05 (1.42)	7.10 (2.16)	7.00 (0.5)	6.38 (1.38)	8.00 (1.95)	12.00 (4.36)	7.50	6.10 (2.56)	3.357	3.544*	2.382
TUA (cm^2)	49.52 (8.18)	46.13 (4.23)	45.55 (7.38)	58.94 (19.15)	47.19 (7.89)	40.09 (3.60)	0.296	4.817*	1.964	48.12 (7.05)	47.71 (6.12)	46.08 (5.15)	45.50 (9.68)	47.76 (8.30)	66.46 (21.72)	44.34	40.16 (3.60)	0.643	3.773*	2.916
UMA (cm^2)	40.46 (7.69)	36.85 (3.73)	36.69 (6.83)	50.09 (19.21)	38.68 (7.60)	31.74 (3.54)	0.528	4.812*	1.977	38.80 (6.52)	38.68 (6.50)	37.34 (4.76)	36.72 (8.61)	38.98 (7.87)	57.80 (21.63)	35.65	31.92 (4.15)	0.88	3.878*	3.077*
UFA (cm^2)	9.07 (0.95)	9.28 (0.77)	8.86 (0.68)	8.85 (0.90)	8.51 (1.13)	8.35 (0.62)	3.059	0.560	0.333	9.32 (0.82)	9.03 (0.71)	8.74 (0.56)	8.78 (1.10)	8.78 (0.80)	8.66 (1.22)	8.70	8.23 (0.93)	1.163	0.763	0.08
UFI (%)	18.58 (2.39)	20.16 (1.26)	19.73 (2.19)	16.36 (5.30)	18.31 (3.17)	20.95 (2.34)	1.021	3.158	1.279	19.55 (1.83)	19.24 (3.19)	19.06 (1.42)	19.61 (2.08)	18.69 (2.54)	14.24 (5.74)	19.61	20.72 (3.50)	0.865	2.119	1.755
TCA (cm^2)	101.59 (13.17)	96.07 (9.54)	85.55 (12.30)	112.64 (21.13)	96.73 (16.45)	84.39 (6.17)	0.613	7.984‡	0.735	97.08 (8.15)	100.46 (15.56)	89.02 (3.26)	89.74 (23.43)	101.52 (12.91)	115.43 (32.40)	89.89	84.71 (6.28)	0.424	3.126*	0.649
CMA (cm^2)	76.53 (9.91)	76.62 (9.50)	68.3 (11.24)	78.86 (6.42)	71.97 (9.18)	64.72 (5.62)	0.414	4.556*	0.528	75.79 (8.57)	78.30 (8.11)	68.60 (2.19)	70.14 (18.94)	75.67 (8.77)	74.93 (14.60)	67.18	66.35 (2.65)	0.301	1.674	0.084
CFA (cm^2)	25.06 (5.82)	19.45 (2.72)	17.25 (2.35)	33.78 (16.39)	24.76 (10.01)	19.68 (6.05)	4.378*	6.088‡	0.479	21.29 (4.78)	22.16 (8.03)	20.41 (1.77)	19.60 (5.56)	25.85 (7.81)	40.49 (18.55)	22.71	18.36 (7.19)	3.742	3.623*	2.354
CFI (%)	24.59 (4.09)	20.37 (3.01)	20.40 (2.96)	28.68 (9.24)	25.01 (6.71)	23.16 (6.38)	4.557*	2.815	0.092	21.99 (4.70)	21.59 (4.29)	22.92 (1.42)	22.01 (3.48)	25.25 (5.72)	33.64 (8.30)	25.27	21.31 (6.62)	4.138	1.837	2.158
TTA (cm^2)	178.96 (31.06)	169.44 (31.51)	163.68 (19.65)	218.99 (54.98)	183.95 (41.19)	162.49 (25.59)	2.383	3.307*	1.090	178.77 (15.24)	187.92 (36.18)	143.55 (39.63)	152.88 (19.52)	198.13 (28.57)	231.20 (73.92)	191.16	150.71 (19.04)	4.812*	5.333‡	0.941
TMA (cm^2)	162.81 (31.32)	152.84 (29.35)	147.16 (18.96)	203.89 (57.86)	168.06 (40.72)	147.04 (25.47)	2.629	3.373*	1.087	161.72 (14.31)	171.26 (37.52)	129.32 (37.42)	136.95 (18.73)	181.14 (28.29)	218.82 (75.51)	175.58	135.21 (20.20)	5.070*	5.352‡	1.03
TFA (cm^2)	16.15 (1.27)	16.60 (2.38)	16.52 (1.09)	15.10 (3.35)	15.89 (1.81)	15.45 (1.34)	2.066	0.314	0.033	17.05 (1.39)	16.66 (1.42)	14.23 (2.25)	15.93 (0.83)	16.99 (1.37)	12.38 (1.59)	15.59	15.51 (1.57)	2.124	5.769‡	4.461‡
TFI (%)	9.23 (1.47)	9.92 (0.95)	10.19 (1.04)	7.54 (3.25)	8.92 (1.89)	9.70 (1.74)	3.302	2.466	0.357	9.56 (0.62)	9.18 (2.07)	10.18 (1.46)	10.51 (0.94)	8.69 (1.18)	6.04 (3.21)	8.15	10.48 (1.98)	6.012*	4.763‡	1.547

%F	10.22 (3.38)	8.56 (3.16)	11.16 (2.34)	12.92 (7.18)	12.53 (3.88)	9.78 (2.76)	1.836	0.308	1.474	8.82 (2.82)	9.91 (4.05)	11.61 (3.69)	11.51 (1.74)	10.38 (2.61)	17.02 (6.26)	11.04	10.35 (5.20)	1.397	1.894	1.912
FM (kg)	7.02 (3.11)	5.39 (2.11)	6.40 (2.20)	9.88 (7.18)	6.97 (2.26)	4.87 (1.31)	0.741	2.384	1.290	5.68 (2.23)	6.65 (3.72)	7.37 (2.81)	6.71 (2.19)	6.03 (1.86)	13.41 (7.36)	6.18	5.20 (2.56)	0.793	3.43*	2.826
FFM (kg)	59.88 (6.84)	57.36 (4.37)	50.27 (9.30)	60.22 (9.01)	48.83 (8.12)	45.13 (4.04)	3.558*	9.166†	1.267	57.88 (5.05)	58.35 (6.98)	54.97 (3.04)	51.54 (14.95)	52.05 (7.67)	60.59 (15.40)	49.82	45.40 (4.06)	1.307	2.624	0.492
R (Ω)	503.37 (43.66)	521.28 (49.57)	561.00 (68.51)	521.14 (108.10)	497.66 (115.77)	570.42 (77.72)	0.002	2.032	0.263	518.24 (48.29)	504.62 (26.12)	556.60 (17.94)	543.25 (107.1)	493.48 (116.58)	490.90 (116.95)	564.90	589.52 (53.56)	0.018	1.804	0.448
Xc (Ω)	58.97 (4.27)	62.04 (5.20)	62.63 (8.41)	66.20 (12.54)	59.90 (7.78)	60.98 (4.83)	0.229	0.159	1.677	60.10 (4.21)	57.70 (5.54)	64.97 (3.97)	64.58 (9.25)	63.18 (6.06)	68.30 (16.97)	63.50	57.58 (4.83)	0.22	0.222	2.3
PA	6.72 (0.53)	6.83 (0.58)	6.53 (0.66)	7.70 (3.26)	7.40 (2.93)	6.16 (0.63)	0.519	0.960	0.522	6.67 (0.55)	6.70 (0.58)	6.67 (0.58)	6.85 (0.82)	7.73 (2.43)	8.50 (4.18)	6.40	5.60 (0.47)	0.291	1.289	1.609
R/H ((Ω/cm)	280.19 (27.70)	303.69 (28.04)	341.58 (62.26)	292.63 (59.33)	297.16 (69.83)	348.40 (42.13)	0.072	4.819*	0.132	291.72 (29.33)	294.37 (21.22)	325.23 (12.42)	336.06 (93.64)	295.44 (70.47)	284.74 (80.61)	336.45	345.52 (24.30)	0.033	2.189	0.058
Xc/H ((Ω/cm)	32.82 (2.85)	36.18 (3.39)	38.09 (6.69)	37.20 (7.02)	35.72 (4.52)	37.27 (2.50)	0.455	1.018	1.234	33.83 (2.60)	33.65 (3.59)	37.95 (2.11)	39.80 (8.35)	37.73 (2.77)	39.31 (9.49)	37.82	33.76 (2.35)	0.234	0.241	3.166*
Yo-Yo IRT	2606.67 (471.54)	2542.50 (784.52)	2255.00 (343.85)	1480.00 (613.84)	1480.00 (446.62)	2104.00 (385.33)	14.100†	0.232	2.160	2466.67 (587.88)	2665.00 (644.02)	2200.00 (226.27)	2580.00 (927.15)	1936.00 (294.75)	1413.33 (938.37)	2400.00	1504.00 (398.60)	6.796*	0.241	1.3
CMJ (cm)	37.10 (6.29)	35.95 (5.87)	36.73 (7.33)	29.16 (6.04)	28.76 (4.34)	27.80 (3.92)	16.667†	0.080	0.064	36.40 (7.29)	36.74 (2.08)	39.17 (10.16)	35.08 (3.96)	31.22 (1.37)	25.87 (8.49)	30.40	26.66 (3.50)	12.822†	0.76	0.45
Sprint 15 meters (s)	2.43 (0.14)	2.42 (0.10)	2.37 (0.11)	2.77 (0.15)	2.75 (0.17)	2.77 (0.09)	70.864†	0.150	0.302	2.44 (0.12)	2.37 (0.03)	2.39 (0.23)	2.41 (0.09)	2.69 (0.09)	2.86 (0.15)	2.69	2.81 (0.14)	55.565†	0.537	1.921
RSA (s)	5.67 (0.21)	5.71 (0.20)	5.68 (0.22)	6.10 (0.07)	6.30 (0.23)	6.17 (0.25)	33.834†	0.671	0.247	5.69 (0.19)	5.71 (0.11)	5.62 (0.37)	5.73 (0.23)	6.22 (0.22)	6.14 (0.02)	5.88	6.29 (0.24)	22.497†	1.065	0.408

Note: E, early; OT, on time; L, late; Q1, quartile one; Q2, quartile two; Q3, quartile three; Q4, quartile four; Bo, Bologna F.C.; Ru, Russi: S. U.; MS, maturity status; RAE, relative age effect; SD, standard deviation; F, Snedecor-Fischer statistic test; BMI, body mass index; circ., circumference; SK, skinfold thickness; TUA, total upper area; UMA, upper muscle area; UFA, upper-fat area; UFI, upper-fat index; TCA, total calf area; CMA, calf mass area; CFA, calf fat area; CFI, calf fat index; TTA, total thigh area; TMA, thigh mass area; TFA, thigh fat area; TFI, thigh fat index; %F, fat percentage; FM, fat mass; FFM, fat-free mass; R, resistance; Xc, reactance; PA, phase angle; CMJ, counter-movement jump; RSA, repeated sprint ability; *, p-value ≤ 0.05; ‡, p-value ≤ 0.01; †, p-value ≤ 0.001.
